# Supplementary material for: Revisiting the specificity and ability of phospho-S129 antibodies to capture alpha-synuclein biochemical and pathological diversity
Source: NPJ Parkinsons Dis. 2022 Oct 20;8:136. doi: 10.1038/s41531-022-00388-7 (PMC9584898; doi:10.1038/s41531-022-00388-7)
Supplement: Supplementary file 1 — Supplementary data [file 41531_2022_388_MOESM1_ESM.pdf]

Supplemental Information

Revisiting the specificity and ability of phospho-S129 antibodies to capture alpha-synuclein biochemical and pathological diversity

Hilal A. Lashuel[1*#], Anne-Laure Mahul-Mellier[1#], Salvatore Novello[1#], Ramanath Narayana Hegde[1], Yllza Jasiqi[1], Melek Firat Altay[1], Sonia Donzelli[1], Sean M. DeGuire[1], Ritwik Burai[1], Pedro Magalhães[1], Anass Chiki[1], Jonathan Ricci[1], Manel Boussouf[1], Ahmed Sadek[1], Erik Stoops[4], Christian Iseli[2,3] and Nicolas Guex[2,3]

Affiliations:

[1]Laboratory of Molecular and Chemical Biology of Neurodegeneration, School of Life Sciences, Brain Mind Institute, Ecole Polytechnique Fédérale de Lausanne (EPFL), CH-1015 Lausanne, Switzerland

[2]Bioinformatics Competence Center, Ecole Polytechnique Fédérale de Lausanne, CH-1015 Lausanne, Switzerland

[3]Bioinformatics Competence Center, University of Lausanne, CH-1015 Lausanne, Switzerland

[4]ADx NeuroSciences, Technologiepark 94, Ghent, Belgium

* Corresponding author: Hilal A. Lashuel, Email: hilal.lashuel@epfl.ch

Running title: Revisiting the specificity of pS129 antibodies

Keywords: Synuclein, phosphorylation, antibodies, aggregation, posttranslational modifications.

Supplementary Figure 1

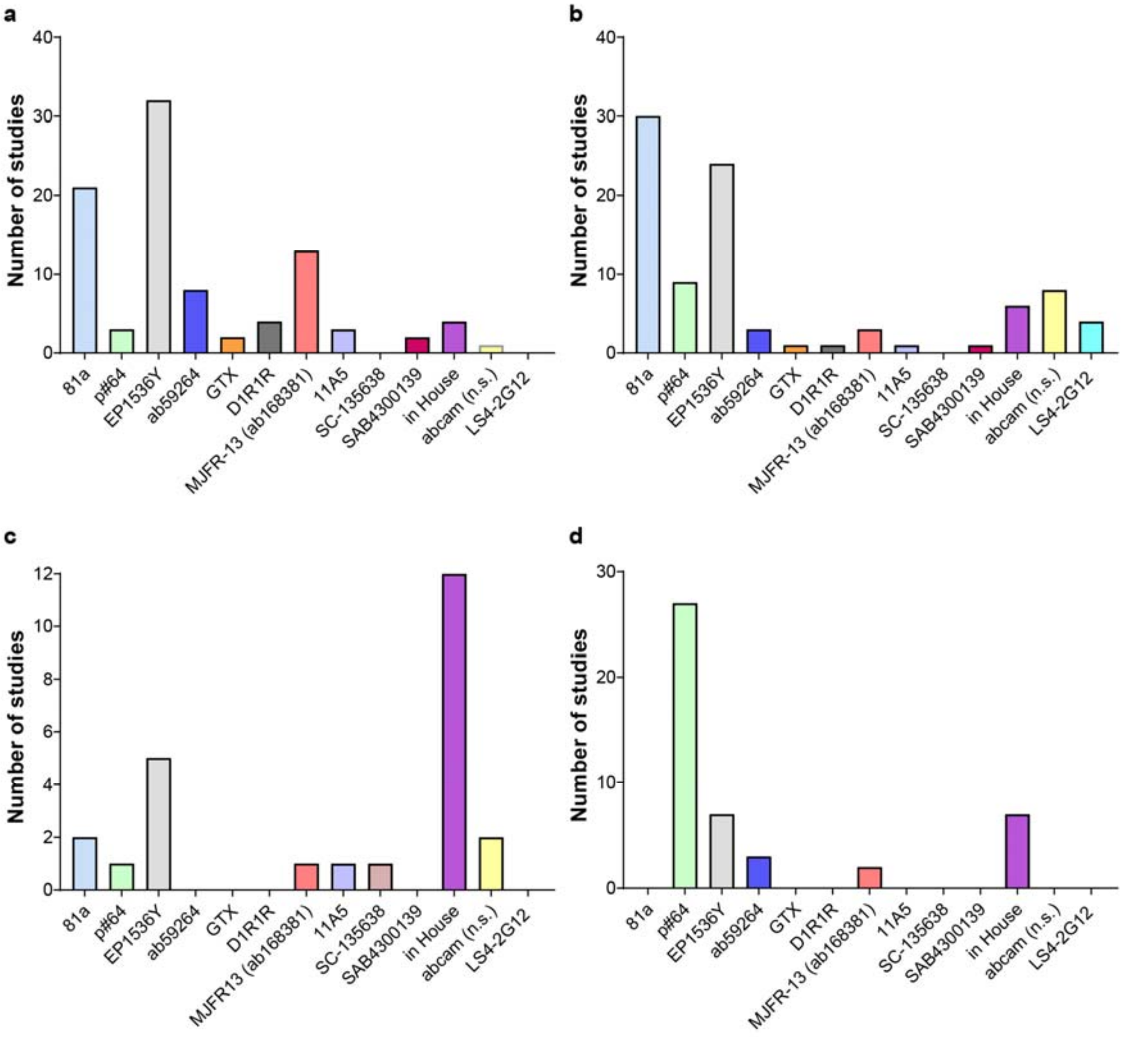

Supplementary Figure 1

e. Seeding model cell culture studies

References	Year of publication	pS129 antibody(-ies) used
Luk et al.	2009	81A
Volpicelli-Daley et al.	2011	81A
Tanik et al.	2013	81A
Aulic et al.	2014	MJF-R13 (ab168381);EP1536Y; ab59264
Tran et al.	2014	81A
Volpicelli-Daley et al.	2014	81A; EP1536Y
Ma et al.	2016	MJF-R13 (ab168381);EP1536Y; ab59264
Mao et al.	2016	81A; MJF-R13 (ab168381)
Samuel et al.	2016	11A5
Abdelmotilib et al.	2017	81A
Karampetsou et al.	2017	p#64, EP1536Y
Karpowicz et al.	2017	81A; in-house
Tapias et al.	2017	EP1536Y
Froula et al.	2018	81A; EP1536Y
Grassi et al.	2018	MJF-R13 (ab168381); GTX
Henderson et al.	2018	MJF-R13 (ab168381); in-house
Mahul-Mellier et al.	2018	81A; MJF-R13 (ab168381)
Peng et al.	2018	81A
Polinski et al.	2018	81A
Sorrentino et al.	2018	EP1536Y
Terada et al.	2018	p#64; EP1536Y
Wang et al.	2018	81A; MJF-R13 (ab168381); EP1536Y
Chen et al.	2019	anti-pS129 (Abcam) (n.s)
Elfarrash et al.	2019	D1R1R;11A5
Froula et al.	2019	EP1536Y
Gao et al.	2019	MJF-R13 (ab168381); EP1536Y
Grassi et al.	2019	GTX
Gribaudo et al.	2019	81A; EP1536Y; ab59264
McGlinchey et al.	2019	ab59264
Volpicelli-Daley et al.	2019	EP1536Y
Wang et al.	2019	EP1536Y
Wu et al.	2019	81A
Ardah et al.	2020	EP1536Y
Bengoa-Vergniory et al.	2020	EP1536Y
Challis et al.	2020	ab59264
Courte et al.	2020	EP1536Y; ab59264; D1R1R
Gegg et al.	2020	EP1536Y
Guan et al.	2020	p#64; in-house
Henderson et al.	2020	MJF-R13 (ab168381); EP1536Y
Henrich et al.	2020	EP1536Y
Mahul-Mellier et al.	2020	81A; MJF-R13 (ab168381); SAB4300139
Morgan et al.	2020	EP1536Y
Apicco et al.	2021	EP1536Y
Burbidge et al.	2021	MJFR13 (ab168381)
Courte et al.	2021	EP1536Y; ab59264; D1R1R
Elfarrash et al.	2021	D1R1R;11A5
Emmenegger et al.	2021	81A; EP1536Y
Mahul-Mellier et al.	2021	81A; MJF-R13 (ab168381); SAB4300139
Marotta et al.	2021	81A
Pantazopoulou et al.	2021	EP1536Y
Stykel et al.	2021	81A; EP1536Y
Tanudjojo et al.	2021	EP1536Y; in-house
Trinkaus et al.	2021	EP1536Y
Vajhøj et al.	2021	EP1536Y
Zhang et al.	2021	EP1536Y
Awa et al.	2022	81A; EP1536Y
Vasili et al.	2022	MJF-R13 (ab168381);EP1536Y; ab59264

Supplementary Figure 1

f. in vivo and mouse strains studies

References	Year of publication	pS129 antibody(-ies) used
Luk et al.	2012a	In-house (pSyn 6.2); in-house (SynpSer129)
Luk et al.	2012b	In-house (pSyn 6.2); in-house (SynpSer129)
Masuda-Suzukake et al.	2013	p#64 , in house (1175 provided by Akiyama)
Guo et al.	2013	81A
Tran et al.	2014	81A
Sacino et al.	2014A	81A
Sacino et al.	2014B	81A
Sacino et al.	2014C	81A
Osterberg et al.	2015	81A
Paumier et al.	2015	81A
Mao et al.	2016	MJF-R13 (ab168381); SAB4300139; 81A
Abdelmotilib et al.	2017	81A
Harms et al.	2017	81A
Sorrentino et al.	2017	81A; EP1536Y
Karampetsou et al.	2017	not specified; in-house
Duffy et al.	2018	81A
Luna et al.	2018	81A
milanese et al.	2018	ab59264
Uemura et al.	2018	81A, p#64, EP1536Y
Yun et al.	2018	81A; MJF-R13 (ab168381)
Ayers et al.	2018	LS4-2G12
Sorrentino et al..	2018	LS4-2G12
Okuzumi et al.	2018	ab59264
Rey et al.	2018	EP1536Y
Lee et al.	2018	MJF-R13 (ab168381)
Manfredsson et al.	2018	81A
Ren et al..	2019	anti-pS129 (Abcam) (n.s)
Durante et al.	2019	EP1536Y
Mavroeidi et al.	2019	EP1536Y; 11A5; LS4-2G12
Patterson et al.	2019	81A
Earls et al.	2019	EP1536Y
Chaterjee et al.	2019	EP1536Y
Bieri et al.	2019	EP1536Y
Yabuki et al.	2020	anti-pS129 (Abcam) (n.s); p#64
Zhang et al.	2020	p#64
Jia et al.	2020	EP1536Y
Kim et al.	2020	81A
Kim et al.	2020	EP1536Y
Creed et al.	2020	81A
Schaser et al.	2020	81A; EP1536Y
Ham et al.	2020	p#64
Wu et al.	2020	81A
Howe et al.	2021	MJFR13 (ab168381); GTX50222
Underwood et al.	2021	EP1536Y
Xu et al.	2021	anti-pS129 (Abcam) (n.s)
Bergkvist et al.	2021	EP1536Y
Ferreira et al.	2021	81A, EP1536Y, and LS4-2G12
Marotta et al.	2021	EP1536Y
Sykioti et al.	2021	EP1536Y
Demmings et al.	2021	anti-pS129 (Abcam) (n.s)
Lai et al.	2021	EP1536Y
Kweon et al.	2021	anti-pS129 (Abcam) (n.s)
Zhang et al.	2021	p#64
Dutta et al.	2021	anti-pS129 (Abcam) (n.s)
Lee et al.	2021	81A
Ghosh et al.	2021	81A; ab59264
Apicco et al.	2021	EP1536Y
Kuan et al.	2021	81A
Zhang et al.	2021	EP1536Y
George et al.	2021	anti-pS129 (Abcam) (n.s)
Cole et al.	2021	81A
Gentzel et al.	2021	p#64
Ma et al.	2021	81A
Thomsen etal	2021	D1R1R
Jimenez-ferrer et al.	2021	EP1536Y
Arkan et al.	2021	EP1536Y
Pantayopoulou et al.	2021	EP1536Y
Merghani et al.	2021	anti-pS129 (Abcam) (n.s)
Szego et al.	2021	p#64
Boutros et al.	2021	EP1536Y
Okuda et al.	2022	p#64; EP1536Y
Thakur et al.	2017	81A; pSyn 5038 (Ser129) EMD (Millipore)*
Kakoty 2021	2021	LB509 (ab27766) *

Supplementary Figure 1

g. Bodyfluids biomarker-based studies

References	Year of publication	pS129 antibody(-ies) used
Foulds et al.	2011	EP1536Y; in house
Wang Y et al.	2012	anti-pS129 (Abcam) (n.s); in house
Foulds et al.	2012	EP1536Y; in house
Foulds et al.	2013	EP1536Y
Stewart et al.	2015	In house
Majbour et al.	2016a	In house
Majbour et al.	2016b	In house
van Steenoven et al.	2018	In house
Cariulo et al.	2019	MJF-R13 (8-8) (ab168381)
Lin et al.	2019	81A
Elhadi et al.	2019	pSyn#64 (WAKO)
Tian et al.	2019	81A
Oosterveld et al.	2020	In house
Majbour et al.	2020	In house
Constantinides et al.	2020	In house
Li et al.	2020	sc-135638
Majbour et al.	2021	In house
Schulz et al.	2021	PRTA-11A5; PRTA-23E8
Chen et al.	2021	Anti-pS129 (Abcam) (n.s.)

h. Gastrointestinal studies

References	Year of publication	pS129 antibody(-ies) used
Lebouvier et al.	2008	pSyn#64
Beach et al.	2010	in-house
Lebouvier et al.	2010	pSyn#64
Pouclet et al.	2011	pSyn#64
Boettner et al.	2012	pSyn#64
Pouclet et al.	2012	pSyn#64
Pouclet et al.	2012	pSyn#64
Beach et al.	2013	in-house
Folgoas et al.	2013	pSyn#64
Adler et al.	2014	in-house
Gelpi et al.	2014	pSyn#64
Gray et al.	2014	ab59264
Hilton et al.	2014	pSyn#64
Ito et al.	2014	pSyn#64, in-house
Masuda et al.	2014	pSyn#64
Aldecoa et al.	2015	pSyn#64
Clairembault et al.	2015	pSyn#64
Mu et al.	2015	pSyn#64
Sanchez-Ferro et al.	2015	pSyn#64
Sprenger et al.	2015	pSyn#64
Visanji et al.	2015	ab59264
Adler et al.	2016	in-house
Antunes et al.	2016	ab59264
Beach et al.	2016	in-house
Chung et al.	2016	EP1536Y
Corbille et al.	2016	pSyn#64, in-house
Stokholm et al.	2016	MJF-R13
Vilas et al.	2016	pSyn#64
Barrenschee et al.	2017	pSyn#64
Carletti et al.	2017	pSyn#64
Corbille et al.	2017	pSyn#64
Kim et al.	2017	pS129-aSyn (Santa Cruz)
Rouaud et al.	2017	pSyn#64
Shin et al.	2017	EP1536Y
Beach et al.	2018	MJF-R13
Fernandez-Arcos et al.	2018	pSyn#64
Iranzo et al.	2018	pSyn#64
Killinger et al.	2018	EP1536Y
Lee et al.	2018	EP1536Y
Ruffmann et al.	2018	pSyn#64
Shin et al.	2018	EP1536Y
Fenyi et al.	2019	pSyn#64
Leclair-Visonneau et al.	2019	pSyn#64
Shin et al.	2019	EP1536Y

Supplementary Figure 1. pS129 antibodies used across human studies and animal and cellular seeding models (related to Figures 1 to 8)

a-d. Graphs represent the pS129 antibodies used across the different number of reports in the cell culture studies using the seeding model (**a**), in the animal studies using the seeding model (**b**), in the bodyfluids biomarker-based studies (**c**), and in the gastrointestinal tract (GIT) studies (**d**). In-house corresponds to the pS129 antibodies generated from different individual groups. **e-h.** Tables list the individual studies that used pS129-specific antibodies displayed in the graphs **a-d,** in the cell culture studies using the seeding model (**e**), in the animal studies using the seeding model (**f**), in the bodyfluids biomarker-based studies (**g**) and in GIT studies (**h**). In-house corresponds to the pS129 antibodies generated from different individual groups. Abcam (n.s.): catalog number was not provided in the original manuscript. The red asterisk indicates studies in which total aSyn antibodies have been used to detect pS129 pathology.

Supplementary Figure 2

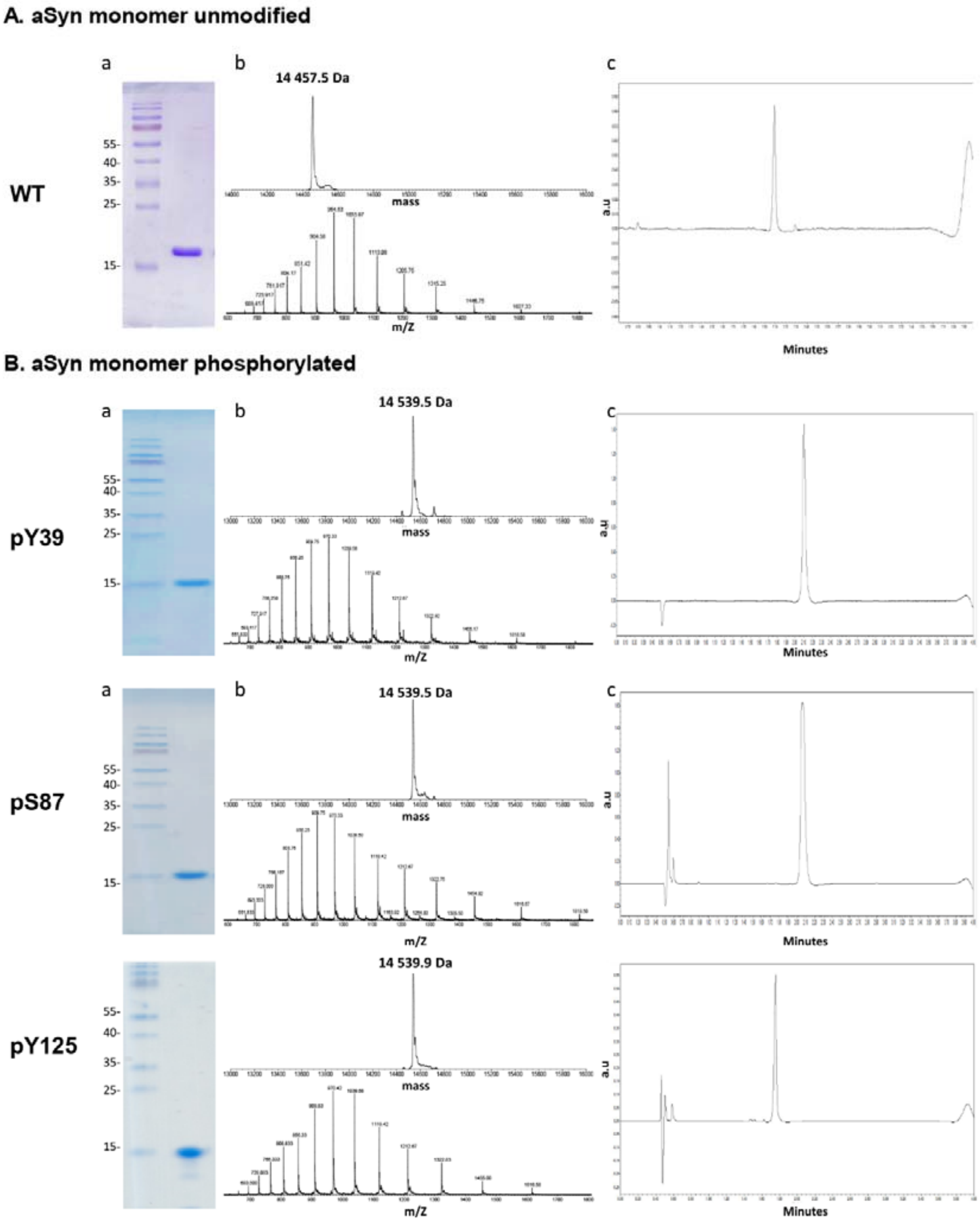

Supplementary Figure 2

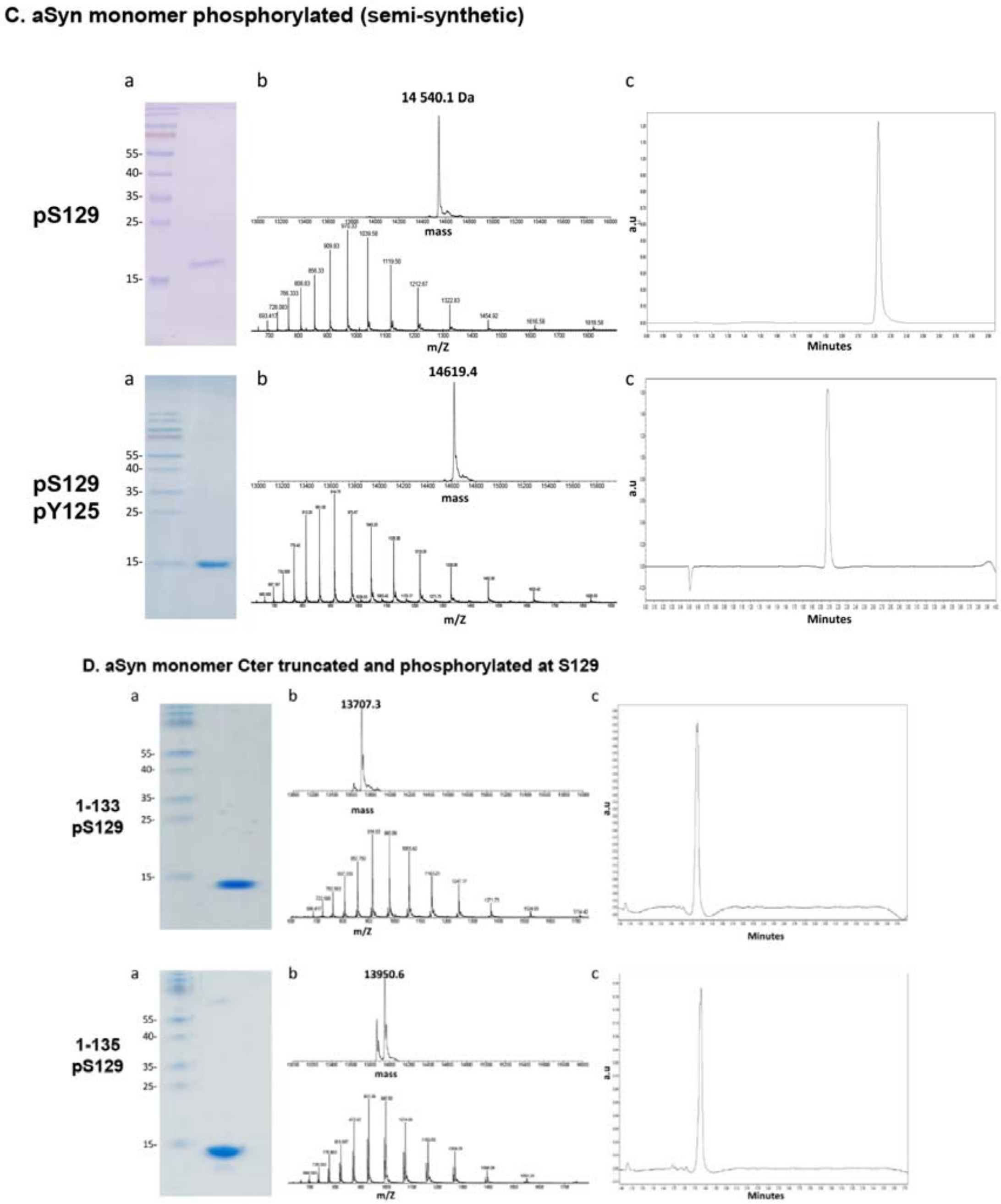

Supplementary Figure 2. Preparation and characterization of recombinant monomeric aSyn species **(related to Figures 1 to 8)**

WT and mutants human aSyn recombinant proteins were produced in *E. coli* and purified by anion exchange chromatography and size-exclusion chromatography, followed by a final chromatographic step using reverse-phase HPLC, as previously described[1]. The purity of recombinant monomeric aSyn was systematically assessed by SDS-PAGE and Coomassie staining (**a**), ESI-LC/MS (**b**), that also showed the expected mass and UPLC (**c**). **A.** Human aSyn unphosphorylated (WT). **B.** Human aSyn phosphorylated at Y39 (pY39), S87 (pS87) or Y125 (pY125). **C.** Human aSyn phosphorylated at Y125 or S129 (pS129) residues or diphosphorylated (pS129 pY125). **D.** Human aSyn C-terminally truncated after the residue 133 (1-133) or 135 (1-135) and phosphorylated at S129.

Supplementary Figure 3

a

Primary Antibody	Catalog #	Company	Clone	RRID	Host/ Clonality	Concentration	WB	ICC	Epitope
anti-aSyn **total**	sc-10717	Santa Cruz	FI-140 (α/β/γ synuclein)	-	Rabbit polyclonal	0.2 mg/mL	1:500	-	**1-140**
anti-aSyn **total**	610787	BD	SYN-1	RRID:AB_398108	Mouse monoclonal	0.25 mg/mL	1:1000	1:1000	**91-99**
anti-aSyn **total**	4179	Cell signalling	D37A6	RRID:AB_1904156	Rabbit monoclonal	1 mg/mL	1:1000	-	**Near Glu105**
anti-aSyn **total**	ab131508	Abcam	-	RRID:AB_11155736	Rabbit polyclonal	1 mg/mL	1:1000	1:500	**134-138**
anti-aSyn **total**	LASHUEL	-	LASH-EGT1-20	-	Rabbit polyclonal	Not provided	1:1000	1:1000	**1-20**
anti-aSyn **total**	LASHUEL	-	LASH-BL-A15110B	-	Mouse monoclonal		1:1000	1:500	**34-45**

b

Primary Antibody	Catalog #	Company	Clone	RRID	Host/ Clonality	Concentration	WB	ICC	HCA	IHC	Epitope
anti-**pS129**-aSyn	825701	BioLegend	p-syn/81A	RRID:AB_2564891	Mouse monoclonal	1.0 mg/mL	1:1000	1:2000	1:1000	1:10000	Peptide (residues 124-134) including phosphorylated Ser129 of human aSyn
anti-**pS129**-aSyn	015-25191	Wako	pSyn #64	RRID:AB_2537218	Mouse monoclonal	1.0 mg/mL	1:1000	1:2000	1:1000	1:1000	residues 124-134
anti-**pS129**-aSyn	ab168381	Abcam	MJF-R13	RRID:AB_2728613	Rabbit monoclonal	4.229 mg/mL	1:3000	1:3000	1:1000	1:2000	The exact sequence is proprietary
anti-**pS129**-aSyn	ab51253	Abcam	EP1536Y	RRID:AB_869973	Rabbit monoclonal	2.578 mg/mL	1:500	1:500*	1:500*	1:1000	Synthetic peptide within human aSyn (aa 100 to the C-terminus). The exact sequence is proprietary.
anti-**pS129**-aSyn	GTX82738	GeneTex	-	AB_11176838	Rabbit polyclonal	1 mg/mL	1:500	1:1000	1:1000	1:1000	Synthetic phospho-peptide corresponding to amino acid residues surrounding Ser129 conjugated to KLH
anti-**pS129**-aSyn	Lashuel	-	pS129-LASH-EGT	-	Rabbit polyclonal	0.1 mg/mL	1:500	1:500	1:500	1:500	A-Y-E-M-P-**SP**-E-E-G-Y-Q-D
anti-**pS129**-aSyn	ab59264	Abcam	-	AB_2270761	Rabbit polyclonal	1 mg/mL	1:500	1:500	1:500	1:1000	Synthetic phosphopeptide derived from human aSyn around the S129 residue (M-P-**S**P-E-E).
anti-**pY125**-aSyn	558246	BD	-	AB_647301	Mouse monoclonal	0.1 mg/mL	1:1000	-	-	-	P-D-N-E-A-**Y**P-E-M-P-S-E-E-G
anti-**pY125**-aSyn	Lashuel	-	pY125-LASH-EGT		Rabbit polyclonal	0.1 mg/mL	1:500	-	-	-	Phosphorylated peptide corresponding to the region including Y125 residue of aSyn
anti-**pY39**-aSyn	849202	Biolegend	A15119B	AB_2650702	Mouse monoclonal	0.5 mg/mL	1:1000	-	-	-	KLH-conjugated α-synuclein synthetic peptide containing pY39
anti-**pS87**-aSyn	Lashuel	-	128005	-	Rabbit polyclonal	0.1 mg/mL	1:1000	-	-	-	H2N- C A Q K T V E G A G S(p) I A A -CONHH

* Due to lot-to-lot variability between 2019 and 2020, the ICC application was removed by Abcam from the EP1536Y antibody datasheet

c

Primary Antibody	Catalog #	Company	Clone	RRID	Host	Concentration	WB dilution	ICC dilution
anti-**actin**	ab6276	Abcam	AC-15	RRID:AB_2223210	Mouse	2.2 mg/mL	1:5000	-
anti-**MAP2**	ab92434	Abcam	-	RRID:AB_2138147	Chicken	Not provided	Not tested	1:2000
anti-**a-Tubulin**	T6199	Sigma	DM1A	RRID:AB_477583	Mouse	Not provided	1:5000	-
anti-**Lamin B1**	ab133741	Abcam	EPR8985	RRID:AB_2616597	Rab Mab	0.33 mg/mL	1:2000	-
anti-**E-Cadherin**	#3195	Cell Signaling	24E10	RRID:AB_2291471	Rab Mab	Not provided	1:1000	-

d

Secondary Antibody	Catalog #	Company	RRID	Concentration	WB dilution	ICC dilution
Goat anti-**mouse Alexa Fluor 680**	A21058	Invitrogen	RRID:AB_2535724	2 mg/mL	1:5000	-
Goat anti-**rabbit Alexa Fluor 680**	A21109	Invitrogen	RRID:AB_2535758	2 mg/mL	1:5000	-
Goat anti-**mouse Alexa Fluor 800**	926-32210	Li-Cor	RRID:AB_621842	1 mg/mL	1:5000	-
Goat anti-**rabbit Alexa Fluor 800**	926-32211	Li-Cor	RRID:AB_621843	1 mg/mL	1:5000	-
Donkey anti-**rabbit Alexa Fluor 647**	A31573	Invitrogen	RRID:AB_2536183	2 mg/mL	-	1:800
Donkey anti-**mouse Alexa Fluor 647**	A31571	Invitrogen	RRID:AB_162542	2 mg/mL	-	1:800
Goat anti-**chicken Alexa Fluor 568**	A11041	Invitrogen	RRID:AB_2534098	2 mg/mL	-	1:500
Goat anti-**mouse Alexa Fluor 488**	A-11029	Invitrogen	RRID:AB_2534088	2 mg/mL	-	1:800
Donkey anti-**chicken Alexa Fluor 488**	703-545-155	Jackson Immunoresearch	RRID:AB_2340375	1 mg/mL	-	1:400
Donkey anti-**rabbit Alexa Fluor 488**	A21206	Invitrogen	RRID:AB_2535792	2 mg/mL	-	1:800

Supplementary Figure 3. List of the antibodies used in this study (related to Figures 1 to 9)

a. Antibodies used for the detection of total aSyn.

b. Antibodies used for the detection of α-syn phosphorylated at residue S129.

c. Other antibodies used in the study.

d. Secondary antibodies used for immunoblotting or confocal imaging.

Supplementary Figure 4

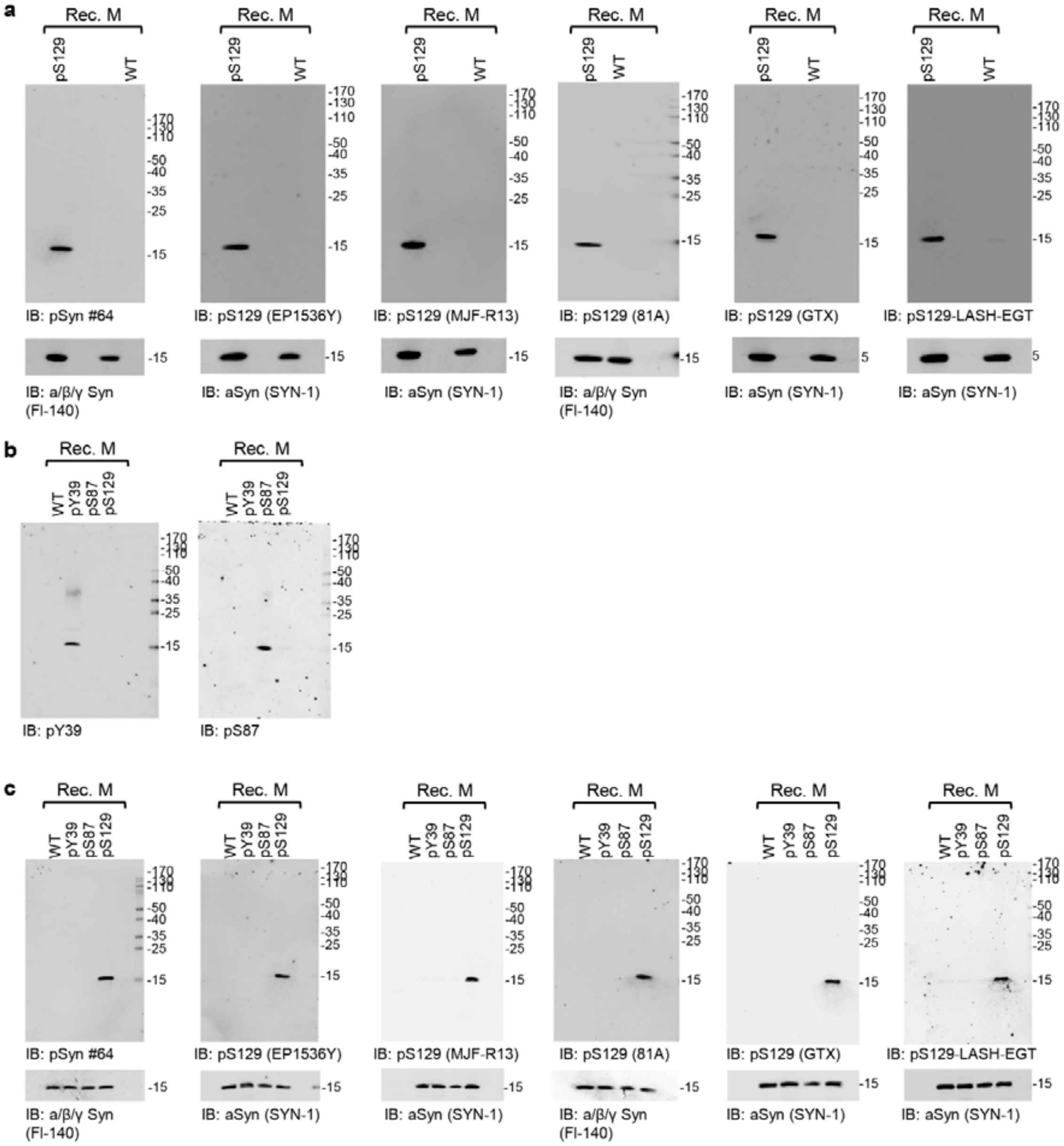

Supplementary Figure 4. The selected pS129 antibodies recognize all specifically pS129-aSyn but not unmodified aSyn (related to Figure 2)

a. Forty nanograms of human recombinant monomeric aSyn unphosphorylated (WT) or phosphorylated at the S129 residue (pS129) were detected by WB using commercial antibodies (pSyn#64, MJF-R13, 81A, EP1536Y and GTX) or the pS129-LASH-EGT homemade pS129 antibody (respective epitopes listed in Figure S3) combined with total aSyn antibodies (SYN-1 or Fl-140 α/β/γ synuclein).

b-c. Forty nanograms of human recombinant monomeric aSyn (WT) or phosphorylated at the Y39 (pY39) or S87 (pS87) or S129 residues (pS129) and WB was performed using the pY39 antibody (**b**) or the pS87-LASH-EGT homemade antibody (**b**) (respective epitopes listed in Figure S3) or the pS129 antibodies (**c**) used in **a** combined with total aSyn antibodies (SYN-1 or Fl-140 α/β/γ synuclein, **c**).

Supplementary Figure 5

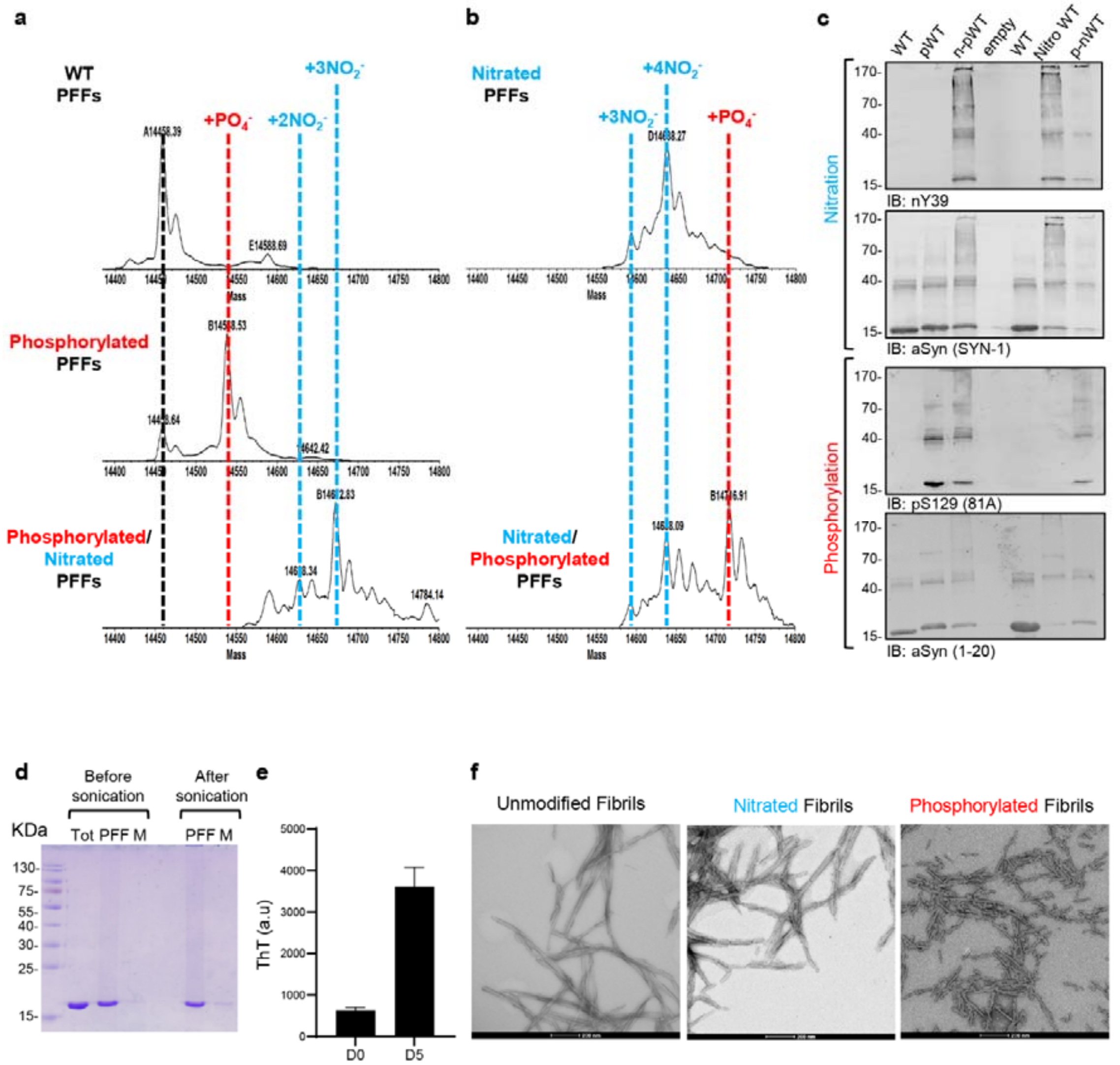

Supplementary Figure 5. Preparation and characterization of recombinant nitrated and/or phosphorylated monomeric and fibrillar aSyn species (related to Figure 3)

a. WT aSyn fibrils were incubated for 18 h at 30°C with the phosphorylating enzyme PLK3. Afterward, the phosphorylated fibrils were incubated for 2 h with the nitric oxide (NO) donor TNM. **b.** WT aSyn fibrils were incubated for 2 h with TNM and afterward incubated for 18 h at 30°C with PLK3. The product reactions were analyzed by ESI-LC/MS. **c.** WB analyses were performed on the samples from **a** and **b**. The presence of the different types of aSyn fibrils was detected by the total aSyn antibody (SYN-1 or 1-20). As expected, the nY39 antibody only detected the nitrated PFFs, while the pS129 antibody (81A) specifically detected the phosphorylated PFFs.

d-f. Characterization of the aSyn PFFs. **d.** The amount of monomer that did not fibrillize or that was released after sonication by SDS-PAGE gel and Coomassie blue staining. **e.** Fibril formation was also confirmed by ThT fluorometry after sonication. All data represent the average ± SD (n=3). **f.** After sonication, aSyn WT, nitrated and/or phosphorylated PFFs were analyzed by transmission electron microscopy (TEM) imaging. Scale bars = 200 nm.

Supplementary Figure 6

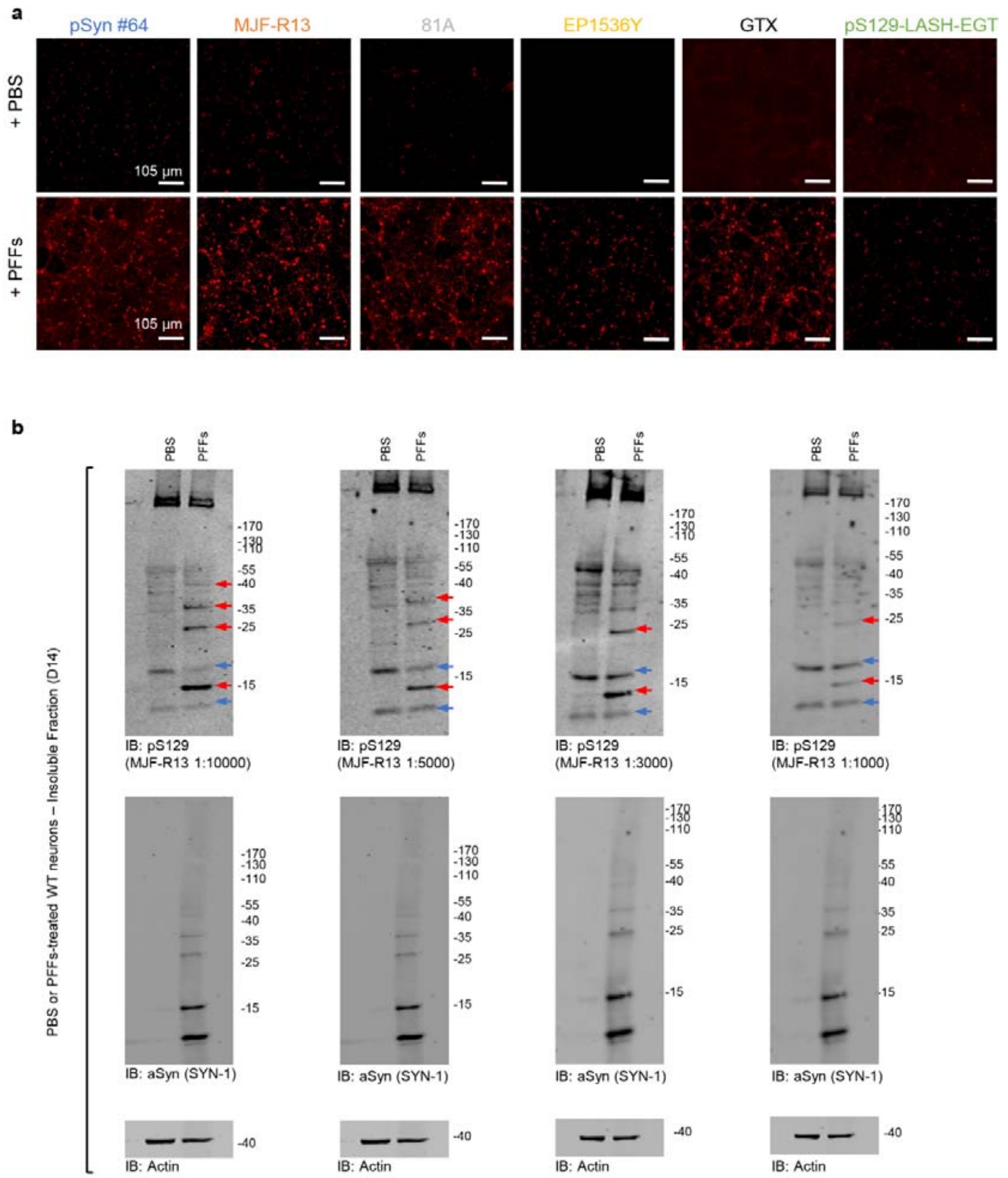

Supplementary Figure 6. Assessment of the pS129 antibodies detection in the PFFs-seeded WT primary hippocampal neurons **(related to Figure 4)**

a. After 14 days of treatment with 70 nM of mouse aSyn PFFs or PBS buffer (negative control), primary hippocampal neurons were fixed, and ICC was performed. Newly formed fibrils were detected using the commercial antibodies (pSyn#64, MJF-R13, 81A, EP1536Y and GTX) or the pS129-LASH-EGT homemade pS129 antibody. Neurons were counterstained with MAP2 antibody and the nucleus with DAPI staining. Images were acquired by a high-throughput wide-field cell imaging system (In Cell Analyser 2200), and pS129 intensity in MAP2-positive neurons was quantified (see quantification in **Figure 4b-c**). Scale bar = 105 µM. **b.** Optimization of the dilution of the MJF-R13 pS129 antibody on the insoluble fractions (SDS-soluble fraction) of primary hippocampal neurons treated with PBS or with 70 nM of mouse aSyn PFFs for 14 days (D14). The optimal concentration of the MJF-R13 antibody is at 1/10 000 for the lot GR3227218 (Abcam). The red arrows indicate the pS129-aSyn positive bands. The blue arrows indicate the undefined bands.

Supplementary Figure 7

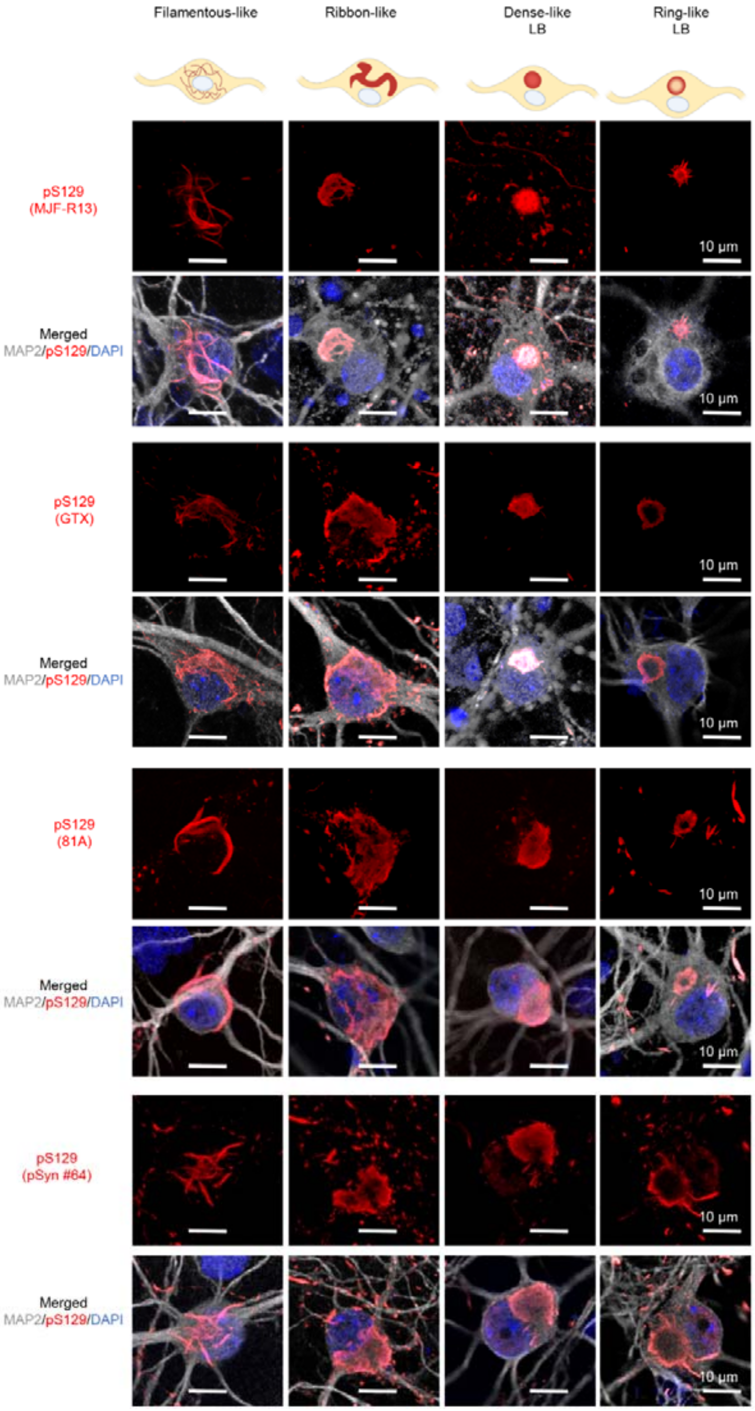

Supplementary Figure 7. The ability of the pS129 antibodies to detect the different types of seeded aggregates in human or mouse PFFs-treated cortical neurons (related to Figure 5)
After 21 days of treatment with 70 nM of mouse aSyn PFFs, primary cortical neurons were fixed, and ICC was performed. Neurons were counterstained with the MAP2 antibody and the nucleus with DAPI staining. Scale bar = 10 μM. The seeded aggregates were detected using the pS129 antibodies (MJF-R13, GTX, 81A or pSyn#64). All four pS129 antibodies were able to distinguish the seeded-aggregates with the filamentous or ribbon-like morphology and the LB-like inclusions with the dense core and the ring-like morphology. The schematic representation of the different types of seeded aggregates was created using BioRender.com

Supplementary Figure 8

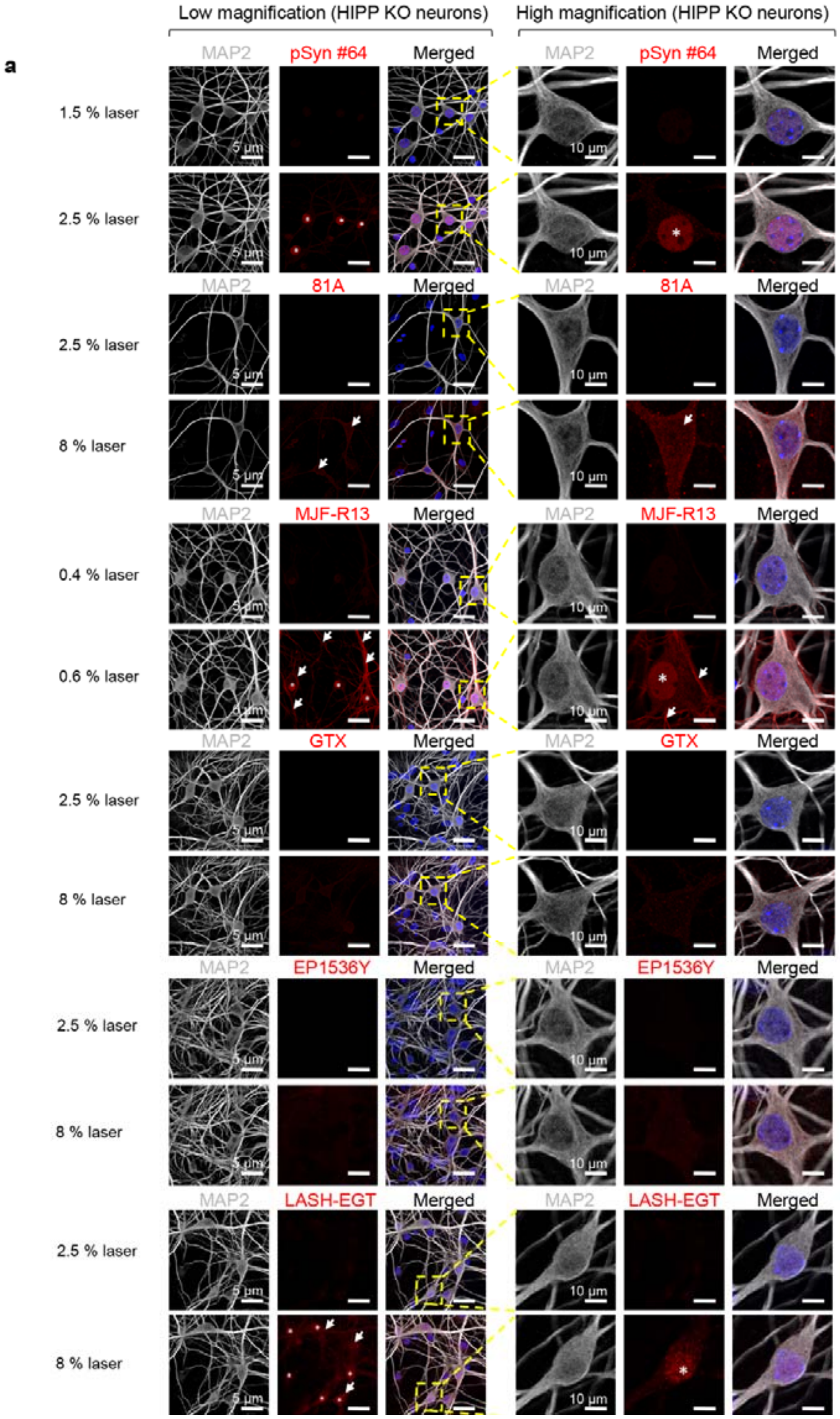

Supplementary Figure 8

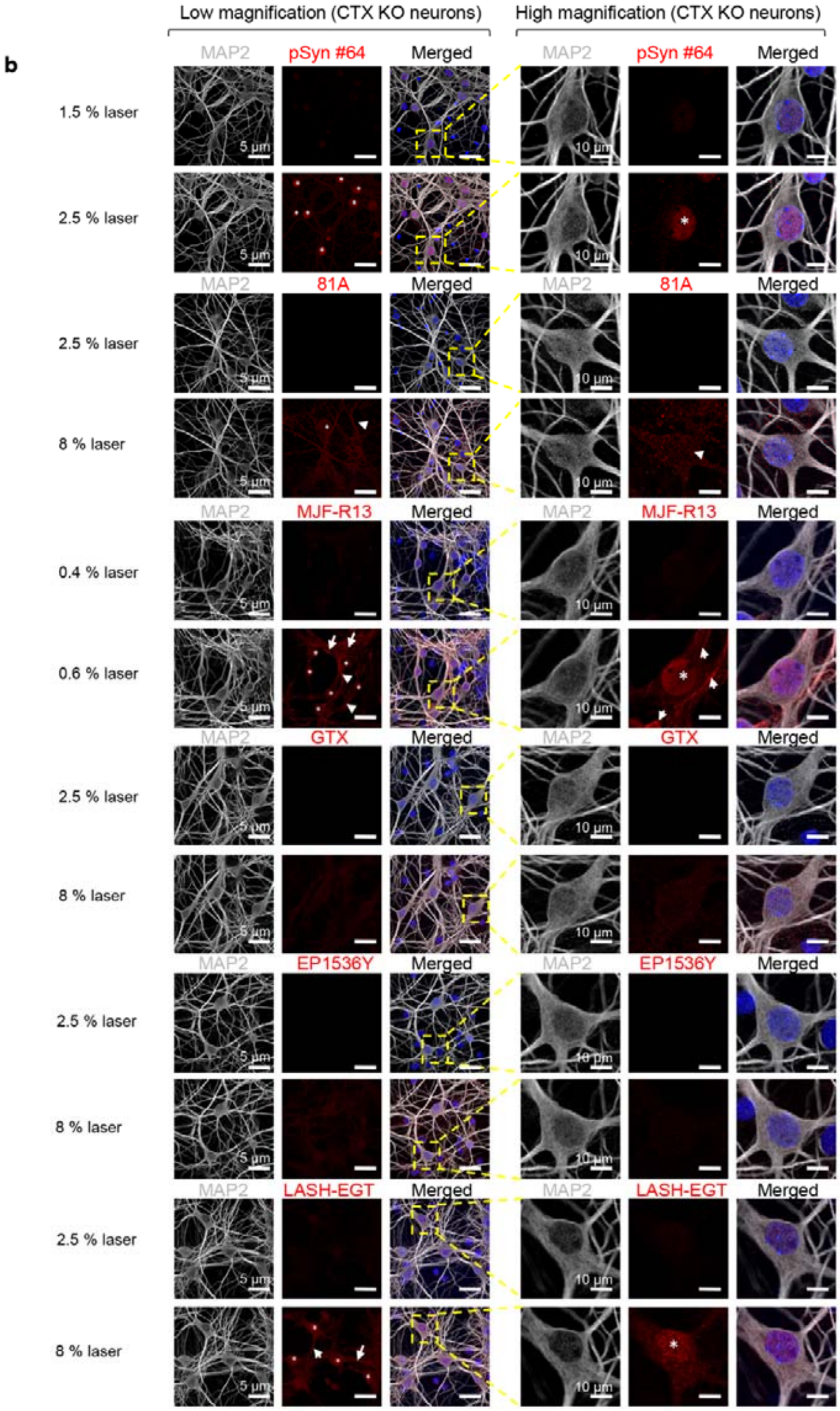

Supplementary Figure 8

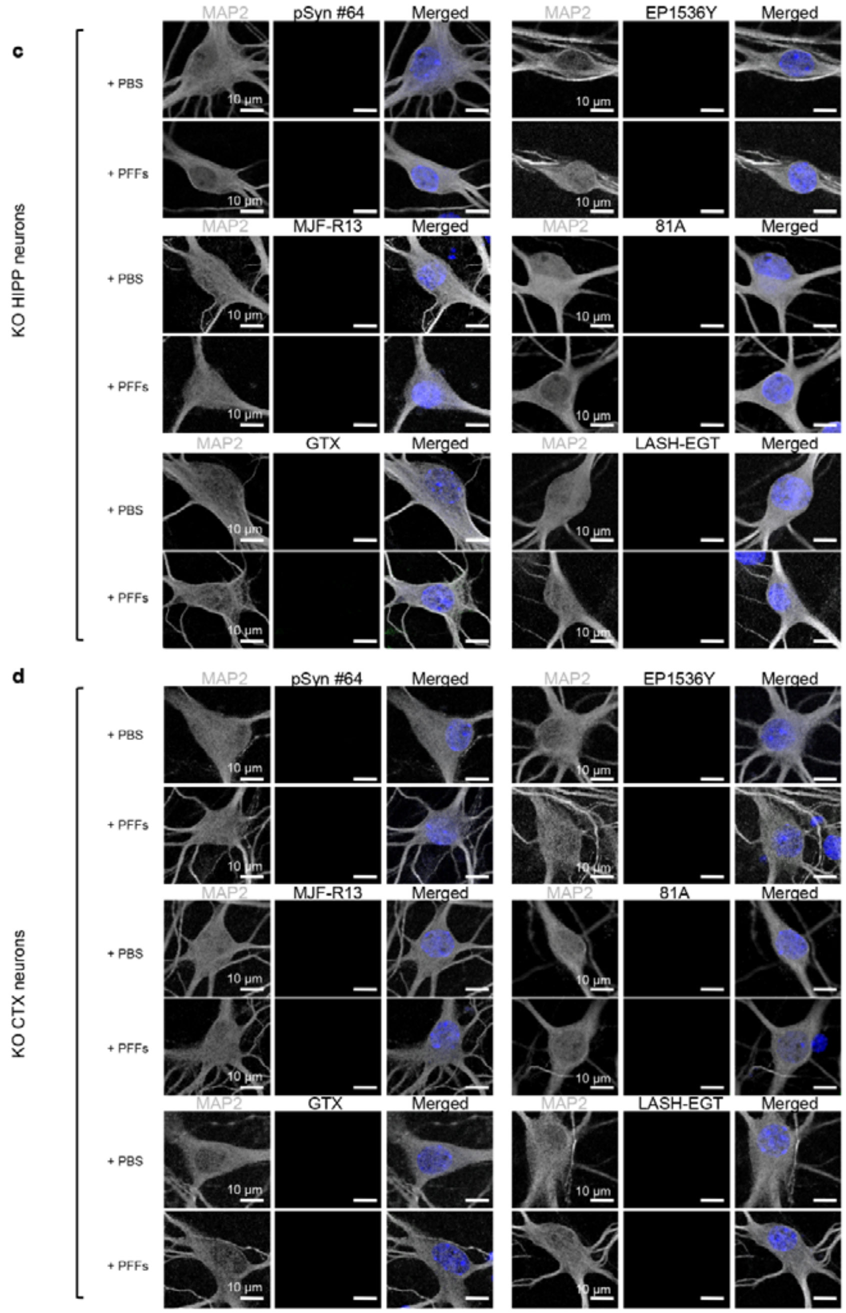

Supplementary Figure 8. Assessment of the pS129 antibodies background and cross-reactivity in aSyn KO primary neurons by ICC and confocal imaging (related to Figures 4-6)

a-b. At DIV14, hippocampal (**a**) or cortical (**b**) aSyn KO primary neurons were fixed, and ICC was performed using the six pS129 antibodies (pSyn#64, MJF-R13, 81A, EP1536Y, GTX and pS129-LASH-EGT). Neurons were counterstained with the MAP2 antibody and the nucleus with DAPI staining. Confocal imaging was performed using different laser powers to reveal the background and the potential cross-reactivity of pS129 antibodies in the nucleus (white star), cytosol and neuritic extensions (white arrows) of neurons lacking aSyn expression. Scale bars = 5 and 10 µM. **c-d.** At DIV14, hippocampal (**c**) or cortical (**d**) aSyn KO primary neurons were treated for 24 h with 70 nM of mouse aSyn PFFs before being fixed. ICC was performed using the six pS129 antibodies (pSyn#64, MJF-R13, 81A, EP1536Y, GTX and pS129-LASH-EGT). Neurons were counterstained with the MAP2 antibody and the nucleus with DAPI staining. Confocal imaging was performed using the same laser intensity and the PMT gain and offset used when imaging WT neurons containing pS129 positive aggregates (Figure 4). This helped eliminate the unspecific signal initially observed in the aSyn KO primary neurons shown in **a** and **b** panels.

Supplementary Figure 9

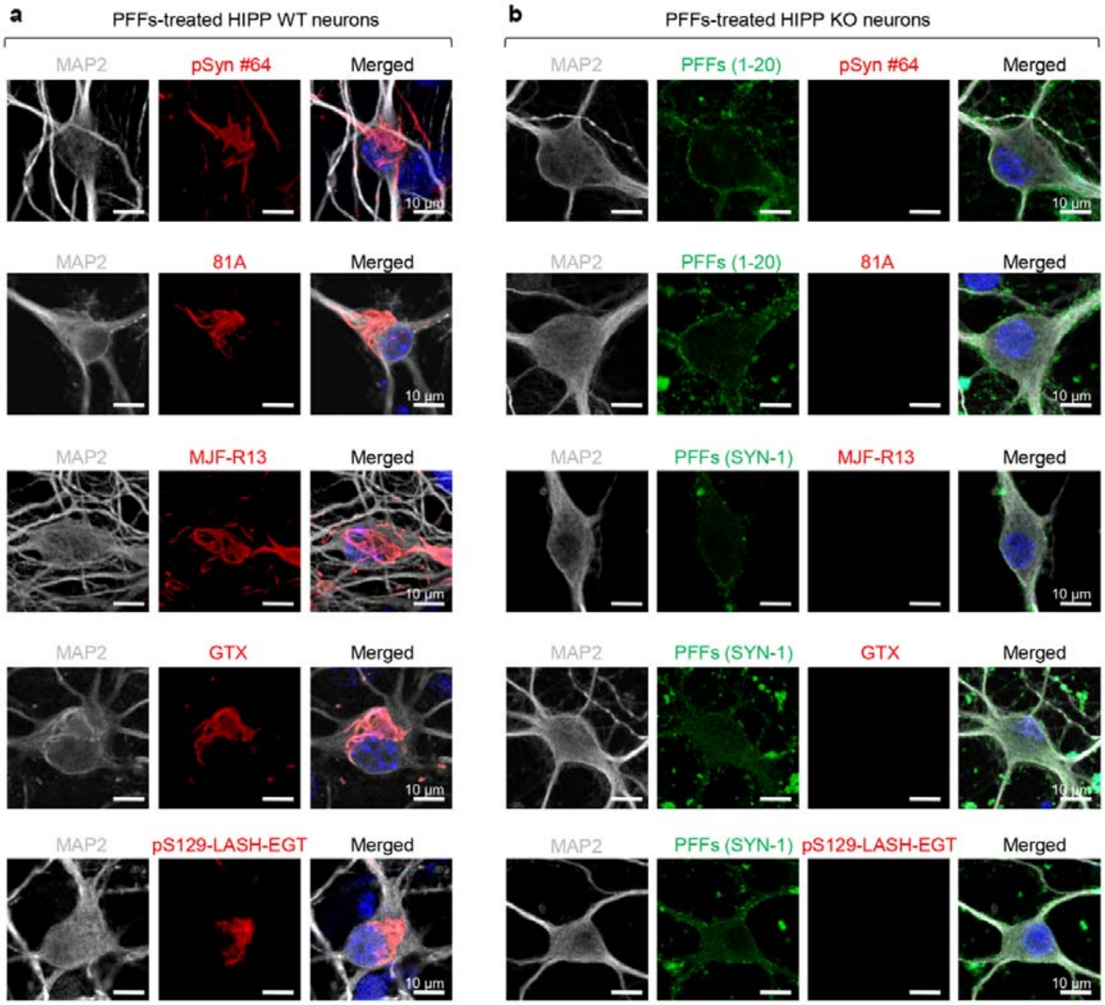

Supplementary Figure 8. Assessment of the pS129 antibodies background and cross-reactivity in aSyn KO and WT primary neurons by ICC and confocal imaging (related to Figures 4-6)
a-b. 70 nM of aSyn PFFs were added for 14 days to the hippocampal WT primary neurons (**a**), and for 24 hours to the aSyn KO primary neurons (**b**). Primary neurons were then fixed, and ICC was performed using the five pS129 antibodies (pSyn#64, MJF-R13, 81A, GTX and pS129-LASH-EGT). EP1536Y was excluded as the recent lot is no longer working by ICC (See Abcam datasheet). Neurons were counterstained with the MAP2 antibody and the nucleus with DAPI staining. In aSyn KO neurons, PFFs were stained with total aSyn antibodies (SYN-1 or 1-20). For each pS129 antibody, the optimal laser power but also PMT gain and offset were set up based on the fluorescence intensity of the seeded aggregates formed in the PFF-treated WT neurons. The same laser intensity, PMT gain and offset were then applied to image the PFF-treated aSyn KO neurons. Under these conditions, the background fluorescence of pS129 in the aSyn KO neurons appears to be negligible.

Supplementary Figure 10

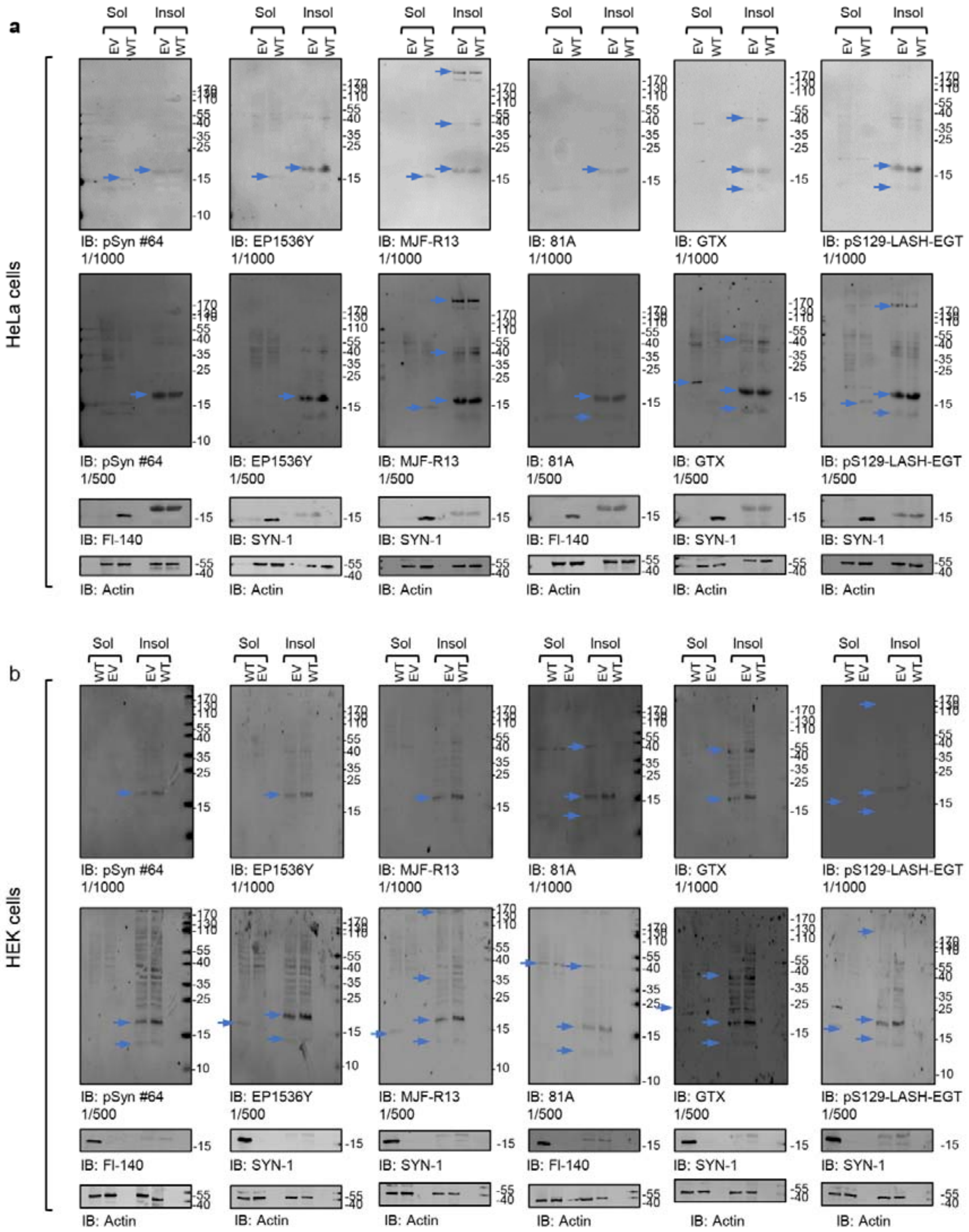

Supplementary Figure 10. Assessment of the pS129 antibodies detection in the soluble and insoluble fractions of Hela and HEK293 mammalian cell lines by WB (related to Figures 6 and 7) HeLa and HEK293 cells were transfected with WT aSyn (WT) or empty (EV) vectors. After 48 h, the transfected cells were lysed and sequential biochemical extraction. pS129 level of detection in the soluble (0.1% Triton-soluble fraction, sol) and the insoluble fraction (SDS-soluble fraction, insol) were extracted from the HeLa (**a**) or HEK293 cells (**b**) using the six pS129 antibodies (pSyn#64, MJF-R13, 81A, EP1536Y, GTX and pS129-LASH-EGT) at a dilution of 1/1000. Membranes were counterstained by total aSyn antibodies (SYN-1 or FI-140 α/β/γ synuclein). Actin was used as a loading control. The red arrows indicate the pS129-aSyn positive bands. The blue arrows indicate the undefined bands.

Supplementary Figure 11

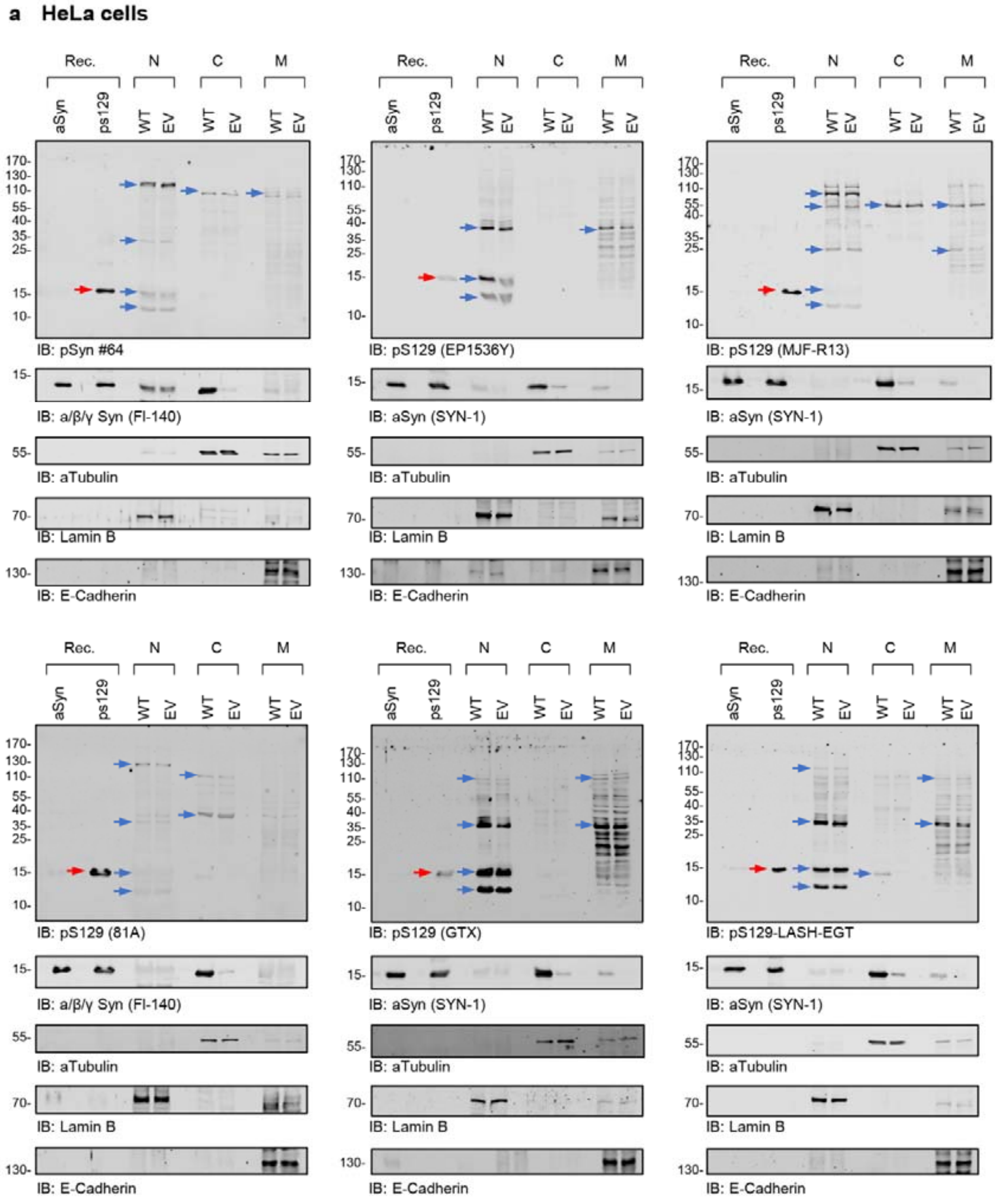

Supplementary Figure 11

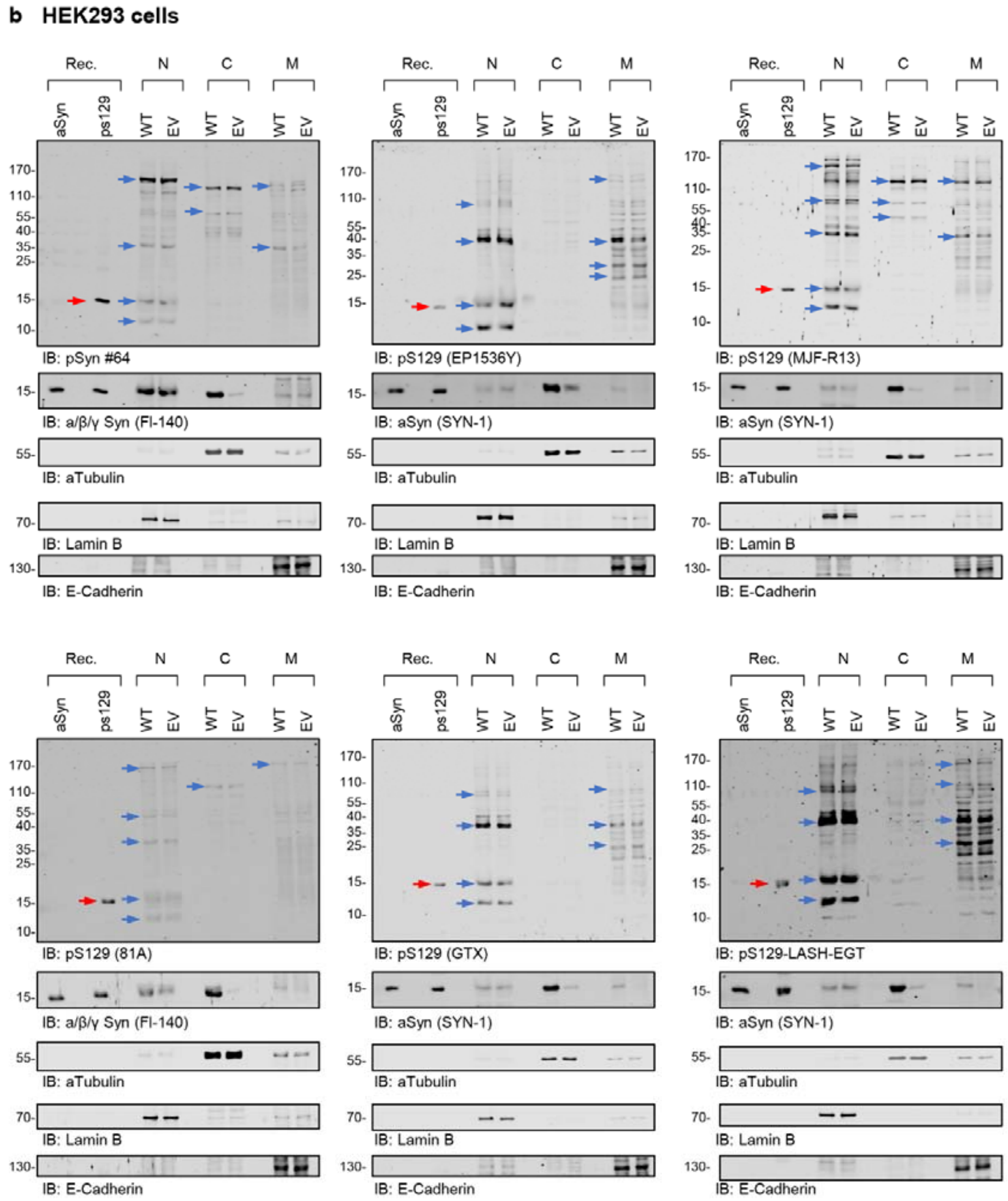

Supplementary Figure 11. Assessment of the pS129 antibodies detection in the nuclear, cytosolic and membranous fractions of Hela and HEK293 mammalian cell lines by WB (related to Figures 6 and 7)

HeLa and HEK293 cells were transfected with WT aSyn or empty (EV) vectors. After 48 h, the transfected cells were lysed and sequential biochemical extraction. pS129 level of detection in the nuclear (N), cytosolic (C), and membrane (M) fractions (A-B) or total cell (C) lysates extracted from the HeLa (**a**) or HEK293 cells (**b**) using the six pS129 antibodies (pSyn#64, MJF-R13, 81A, EP1536Y, GTX and pS129-LASH-EGT) at a dilution of 1/1000. Forty nanograms of recombinant aSyn WT or phosphorylated at residue S129 (pS129, indicated by a red arrow) were used as positive controls. Membranes were counterstained by total aSyn antibodies (SYN-1 or Fl-140 α/β/γ synuclein). aTubulin, Laminin B and E-Cadherin were used as loading control for respectively the cytosolic, nuclear and membrane fractions. The red arrows indicate the pS129-aSyn positive bands. The blue arrows indicate the undefined bands.

Supplementary Figure 12

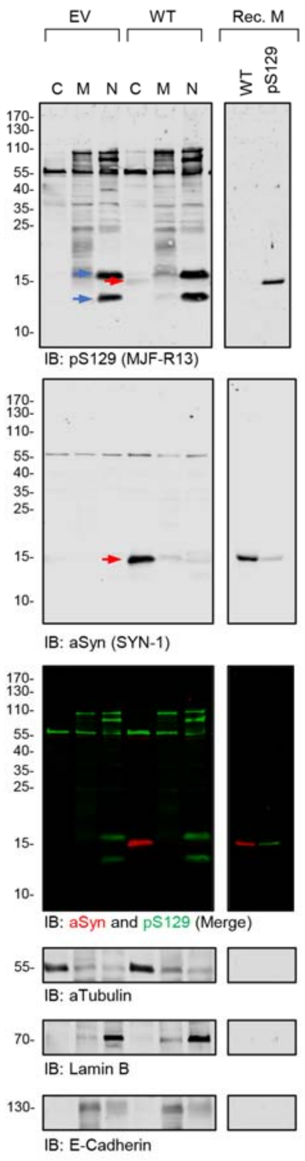

Supplementary Figure 12. The commercial tricine gel allows resolving the aSyn band from the non-specific bands **(related to Figures 4, 6 and 7)**

HeLa cells were transfected with WT aSyn or empty (EV) vectors. After 48 h, the transfected cells were lysed, subcellular fractionation was performed, and proteins from the nuclear (N), cytosolic (C) and membrane (M) fractions were separated using commercial 16% tricine gel (Invitrogen). Forty nanograms of recombinant aSyn WT or phosphorylated at residue S129 (pS129) were used as positive controls. Membranes were co-stained with the MJF-R13 (pS129) and SYN-1 (total aSyn). Actin was used as a loading control. The red arrows indicate the pS129-aSyn positive bands. The blue arrows indicate the undefined bands.

Supplementary Figure 13

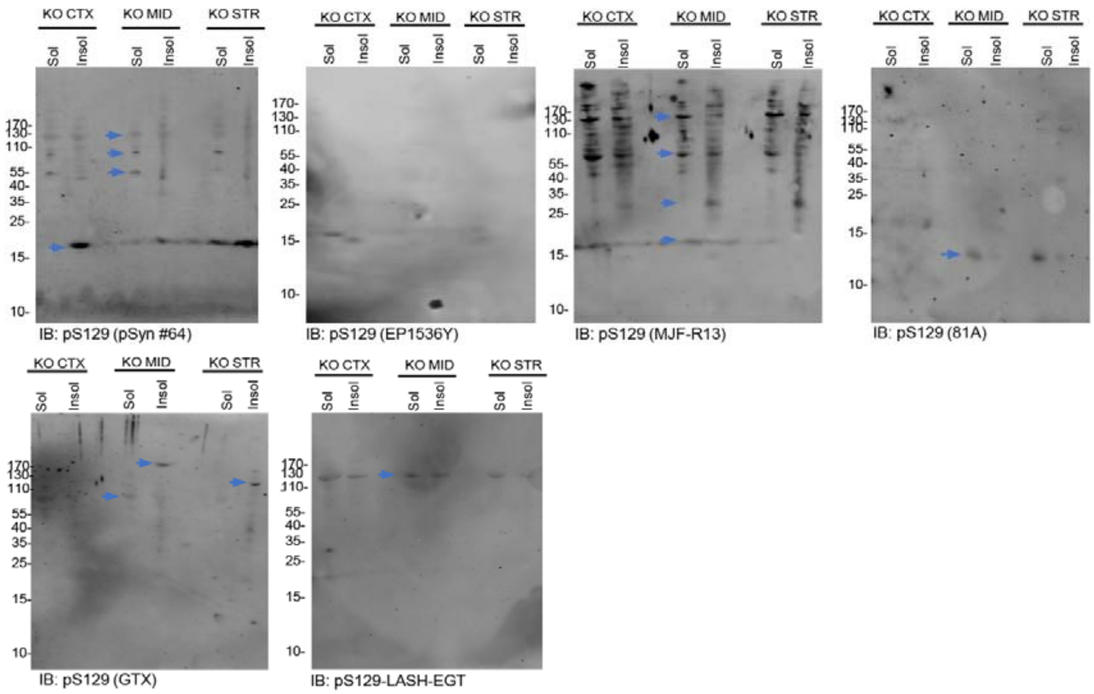

Supplementary Figure 13. Assessment of the pS129 antibodies detection in brain sections of aSyn KO mice (related to Figure 7B)

pS129 level of detection in the soluble and insoluble fractions extracted from the aSyn KO mice brains using the six pS129 antibodies (pSyn#64, MJF-R13, 81a, EP1536Y, GTX and pS129-LASH-EGT). Sequential biochemical extraction was performed on different brain regions (cortical/CTX; Midbrain/MID and Striatum/STR) derived from aSyn KO mice, and the level of pS129 was assessed in the soluble (0.1% Triton-soluble fraction) and the insoluble fraction (SDS-soluble fraction). Membranes were counterstained for total aSyn (SYN-1 or D37A6), and actin was used as a loading control. The red arrows indicate the pS129-aSyn positive bands. The blue arrows indicate the undefined bands. Membranes shown in Figure S12 are the same as in Figure 7B but acquired at a higher exposure.

Supplementary Figure 14

a

	Total	S129	S87	Y39	Y125	Y133	Y136
**Previously detected in plasma**	59	27	13	2	4	2	11
**Previously detected in CSF**	40	19	10	1	3	0	7
**Combined Proteins identified (based on peptide alignment)**	123	59	32	4	5	3	20

b **S129 human – part 1**

Peptide alignment	Position	Length	MW list [kDa]	Exact	Similar	Mismatch	Max exact stretch	Max exact + similar stretch	ID	Also in mouse	Description	Subcellular location	Detected in plasma	Detected in CSF
EAYEMPSEEGYQD<br>:::::::::::::<br>EAYEMPSEEGYQD	123	140	14	13	0	0	13	13	SYUA_HUMAN	Y	Alpha-synuclein	Cell junction. Cytoplasm. Membrane. Nucleus. Secreted	Y	Y
EKVELPSEEQVSG<br>: :.::::<br>EAYEMPSEEGYQD	375	1782	190	6	1	6	4	6	AKA12_HUMAN	Y	A-kinase anchor protein 12	Cytoplasm. Membrane	Y	Y
SFPELPSEE****<br>:.::::<br>EAYEMPSEEGYQD	920	928	100	5	1	7	4	6	NIBA1_HUMAN	Y	Protein Niban 1	Cytoplasm. Membrane	Y	Y
EEDDYPSEELLED<br>: . :::: .:<br>EAYEMPSEEGYQD	721	1907	210	6	2	5	4	4	TGO1_HUMAN	N	Transport and Golgi organization protein 1 homolog	Endoplasmic reticulum membrane	Y	Y
EARKKPSEEEAAV<br>:: ::::<br>EAYEMPSEEGYQD	162	195	21	6	0	7	4	4	CY24A_HUMAN	Y	Cytochrome b-245 light chain	Cell membrane	N	N
KARENPSEEAQNL<br>: : ::::<br>EAYEMPSEEGYQD	115	501	59	6	0	7	4	4	SF3A3_HUMAN	Y	Splicing factor 3A subunit 3	Nucleus. Nucleus speckle	Y	N
EDFRRPSEEDFRR<br>: . :::: .<br>EAYEMPSEEGYQD	585	1001	120	5	2	6	4	4	RB12B_HUMAN	N	RNA-binding protein 12B	unspecified.	N	N
KEELVPSEEDFQG<br>:::: .:<br>EAYEMPSEEGYQD	579	1001	110	5	1	7	4	4	BRE1B_HUMAN	N	E3 ubiquitin-protein ligase BRE1B	Nucleus	N	Y
PARTPPSEEDSAE<br>: :::: .<br>EAYEMPSEEGYQD	78	313	34	5	1	7	4	4	SGTA_HUMAN	Y	Small glutamine-rich tetratricopeptide repeat-containing protein alpha	Cytoplasm. Nucleus	Y	N
VGVEEPSEEDQNE<br>: :::: .<br>EAYEMPSEEGYQD	539	725	80	5	1	7	4	4	SL9A7_HUMAN	N	Sodium/hydrogen exchanger 7	Cell membrane. Golgi apparatus. Recycling endosome membrane	N	N
LPEVPPSEEEEQE<br>:::: :.<br>EAYEMPSEEGYQD	809	1127	130	5	1	7	4	4	TEX2_HUMAN	Y	Testis-expressed protein 2	Endoplasmic reticulum membrane. Nucleus membrane	N	N
KTERKPSEEEYVI<br>:::: :<br>EAYEMPSEEGYQD	634	776	88	5	0	8	4	4	ARHG6_HUMAN	N	Rho guanine nucleotide exchange factor 6	Cell projection	N	N
TKNEEPSEEEIDA<br>: ::::<br>EAYEMPSEEGYQD	115	783	87	5	0	8	4	4	DDX21_HUMAN	Y	Nucleolar RNA helicase 2	Cytoplasm. Mitochondrion. Nucleus	Y	N
PRESRPSEEREWD<br>:::: :<br>EAYEMPSEEGYQD	1192	1382	170	5	0	8	4	4	EIF3A_HUMAN	Y	Eukaryotic translation initiation factor 3 subunit A	Cytoplasm	Y	N
PPAGRPSEEPEPD<br>:::: :<br>EAYEMPSEEGYQD	374	432	47	5	0	8	4	4	PHAG1_HUMAN	Y	Phosphoprotein associated with glycosphingolipid-enriched microdomains 1	Cell membrane	Y*	Y
KIQRMPSESAAQS<br>:::: :<br>EAYEMPSEEGYQD	113	514	57	5	0	8	4	4	PLRG1_HUMAN	Y	Pleiotropic regulator 1	Nucleus. Nucleus speckle	N	N
RGEDVPSEEEEEE<br>. :::: ..<br>EAYEMPSEEGYQD	178	412	47	4	3	6	4	4	NOB1_HUMAN	N	RNA-binding protein NOB1	Nucleus	N	N
SGSQGPSEEKPAP<br>. ::::<br>EAYEMPSEEGYQD	386	1782	190	4	1	8	4	4	AKA12_HUMAN	Y	A-kinase anchor protein 12	Cytoplasm. Membrane	Y	Y
DIIRQPSEEEIIK<br>. ::::<br>EAYEMPSEEGYQD	110	130	15	4	1	8	4	4	PEA15_HUMAN	Y	Astrocytic phosphoprotein PEA-15	Cytoplasm	Y	Y

Supplementary Figure 14

b S129 human – part 2

Peptide alignment	Position	Length	MW list [kDa]	Exact	Similar	Mismatch	Max exact stretch	Max exact + similar stretch	ID	Also in mouse	Description	Subcellular location	Detected in plasma	Detected in CSF
TRKRGPSEETGGA<br>::::<br>EAYEMPSEEGYQD	571	726	81	4	0	9	4	4	CCNT1_HUMAN	N	Cyclin-T1	Nucleus	N	N
FHLMAPSEEDHSI<br>::::<br>EAYEMPSEEGYQD	138	259	28	4	0	9	4	4	IBP1_HUMAN	N	Insulin-like growth factor-binding protein 1	Secreted	Y	Y
LCTKAPSEEDSLS<br>::::<br>EAYEMPSEEGYQD	32	258	29	4	0	9	4	4	RT18B_HUMAN	N	28S ribosomal protein S18b, mitochondrial	Mitochondrion	N	N
KELPPPSEEIKTG<br>::::<br>EAYEMPSEEGYQD	531	657	76	4	0	9	4	4	THOC1_HUMAN	Y	THO complex subunit 1	Cytoplasm, Nucleus, Nucleus matrix and Nucleus speckle	N	N
AMLHLPSEQGAPE<br>.::::.: .<br>EAYEMPSEEGYQD	37	419	48	4	3	6	3	6	NEMO_HUMAN	Y	NF-kappa-B essential modulator	Cytoplasm and Nucleus	Y *	N
EETGSPSEDGMQS<br>: :::.: :<br>EAYEMPSEEGYQD	53	501	56	6	1	6	3	5	PWP1_HUMAN	N	Periodic tryptophan protein 1 homolog	Cytoplasm and Nucleus	N	N
MSRALPSEDEEGQ<br>.::::.<br>EAYEMPSEEGYQD	8299	8797	1000	3	2	8	3	5	SYNE1_HUMAN	Y	Nesprin-1	Cytoplasm, Golgi apparatus, Nucleus, Nucleus envelope, Nucleus outer membrane	Y	Y #
EPEEGPSEDDKAE<br>: : :::. .<br>EAYEMPSEEGYQD	4940	5596	630	5	2	6	3	4	MDN1_HUMAN	N	Midasin	Cytoplasm and Nucleus	N	N
DSSPLPSEKSDED<br>. .::: .:<br>EAYEMPSEEGYQD	1354	1710	200	4	3	6	3	4	CHD1_HUMAN	Y	Chromodomain-helicase-DNA-binding protein 1	Cytoplasm and Nucleus	N	N
ETQRKPSEDEVLN<br>: :::. .<br>EAYEMPSEEGYQD	1052	1231	140	4	2	7	3	4	EHBP1_HUMAN	Y	EH domain-binding protein 1	Cytoplasm, Endosome, Membrane	N	N
YSVQEPSEDSEEE<br>. :::. ..<br>EAYEMPSEEGYQD	1178	1374	140	3	4	6	3	4	LMTK1_HUMAN	Y	Serine/threonine-protein kinase LMTK1	Cytoplasm and Membrane	N	N
QYNQVPSEDFERT<br>. . :::.<br>EAYEMPSEEGYQD	317	365	40	3	3	7	3	4	CXAR_HUMAN	N	Coxsackievirus and adenovirus receptor	Basolateral cell membrane, Cell junction, Cell membrane, Secreted	Y	Y
DSSRLPSEGPRPA<br>. .:::<br>EAYEMPSEEGYQD	36	206	22	3	2	8	3	4	BLVRB_HUMAN	Y	Flavin reductase (NADPH)	Cytoplasm	Y	Y
DQQSLPSEPEETL<br>. .:::<br>EAYEMPSEEGYQD	341	543	58	3	2	8	3	4	RETR2_HUMAN	N	Reticulophagy regulator 2	Membrane	N	Y #
RTQTLPSENSEES<br>.::: .<br>EAYEMPSEEGYQD	54	641	71	3	2	8	3	4	RIOX1_HUMAN	N	Ribosomal oxygenase 1	Nucleus	N	N
KHQSDPSEDEDER<br>:::. .<br>EAYEMPSEEGYQD	123	496	57	3	2	8	3	4	SGK3_HUMAN	Y	Serine/threonine-protein kinase Sgk3	Cytoplasmic vesicle, Early endosome and Recycling endosome	N	N
KSVSTPSEAGSQD<br>::: : ::<br>EAYEMPSEEGYQD	101	645	71;73	6	0	7	3	3	DC1I1_HUMAN	N	Cytoplasmic dynein 1 intermediate chain 1	Chromosome and Cytoplasm	N	N
EEEEEPSEGLEEE<br>: : ::: ..<br>EAYEMPSEEGYQD	335	1035	110	5	2	6	3	3	GGYF1_HUMAN	Y	GRB10-interacting GYF protein 1	Unspecified	N	N
DLKESPSEGSLQP<br>. : ::: :<br>EAYEMPSEEGYQD	2	512	58	5	1	7	3	3	ASIC2_HUMAN	N	Acid-sensing ion channel 2	Cell membrane	N	N

Supplementary Figure 14

b S129 human – part 3

Peptide alignment	Position	Length	MW list [kDa]	Exact	Similar	Mismatch	Max exact stretch	Max exact + similar stretch	ID	Also in mouse	Description	Subcellular location	Detected in plasma	Detected in CSF
EAASGPSESPSPP<br>:: :::<br>EAYEMPSEEGYQD	75	814	92	5	0	8	3	3	EIF3B_HUMAN	N	Eukaryotic translation initiation factor 3 subunit B	Cytoplasm	Y	N
EKNSTPSEPGSGR<br>: ::: :<br>EAYEMPSEEGYQD	196	240	27	5	0	8	3	3	HDGF_HUMAN	N	Hepatoma-derived growth factor	Cytoplasm, Nucleus and Secreted	Y	N
EPDCNPSEAASEE<br>: ::: ..<br>EAYEMPSEEGYQD	158	783	87	4	2	7	3	3	DDX21_HUMAN	Y	Nucleolar RNA helicase 2	Cytoplasm, Mitochondrion and Nucleus	Y	N
TVRYAPSEAGLHE<br>::: : ..<br>EAYEMPSEEGYQD	1829	2647	280	4	2	7	3	3	FLNA_HUMAN	Y	Filamin-A	Cell projection, Cytoplasm and Perikaryon	Y	Y
PAERTPSEIQFHQ<br>: ::: ..<br>EAYEMPSEEGYQD	322	820	92	4	2	7	3	3	M4K2_HUMAN	Y	Mitogen-activated protein kinase kinase kinase kinase 2	Basolateral cell membrane, Cytoplasm and Golgi apparatus membrane	N	N
AKDEPPSEGEAEE<br>: ::: ..<br>EAYEMPSEEGYQD	466	543	62	4	2	7	3	3	NFL_HUMAN	Y	Neurofilament light polypeptide	Cell projection and Cytoplasm	Y	Y
KPGEEPSEYTDEE<br>: ::: ..<br>EAYEMPSEEGYQD	202	223	24	4	2	7	3	3	PGRC2_HUMAN	Y	Membrane-associated progesterone receptor component 2	Endoplasmic reticulum, Membrane and Nucleus envelope	Y	N
KRVRRPSESDKED<br>::: .:<br>EAYEMPSEEGYQD	621	1782	190	4	1	8	3	3	AKA12_HUMAN	Y	A-kinase anchor protein 12	Cytoplasm and Membrane	Y	Y
EDEGIPSENEEEK<br>: ::: .<br>EAYEMPSEEGYQD	2339	2813	310	4	1	8	3	3	AKP13_HUMAN	Y	A-kinase anchor protein 13	Cytoplasm, Membrane and Nucleus	Y	N
HAFSSPSESPDST<br>:. :::<br>EAYEMPSEEGYQD	1159	1429	160	4	1	8	3	3	ANR50_HUMAN	N	Ankyrin repeat domain-containing protein 50	Endosome	N	N
ERLQNPSESSEPI<br>: . :::<br>EAYEMPSEEGYQD	25	185	22	4	1	8	3	3	CASA1_HUMAN	N	Alpha-S1-casein	Secreted	N	N
HGAASPSEKGAHP<br>::: : .<br>EAYEMPSEEGYQD	13	602	66	4	1	8	3	3	CKAP4_HUMAN	N	Cytoskeleton-associated protein 4	Cell membrane, Cytoplasm and Endoplasmic reticulum membrane	Y	N
GSTYTPSEAGNEL<br>::: : .<br>EAYEMPSEEGYQD	208	256	29	4	1	8	3	3	MAF1_HUMAN	N	Repressor of RNA polymerase III transcription MAF1 homolog	Cytoplasm and Nucleus	N	N
DADSEPSESESAS<br>.: :::<br>EAYEMPSEEGYQD	502	814	92	4	1	8	3	3	UBP45_HUMAN	N	Ubiquitin carboxyl-terminal hydrolase 45	Photoreceptor inner segment	N	N
DGLDTPSENSNEF<br>. . ::: .<br>EAYEMPSEEGYQD	83	314	36	3	3	7	3	3	BNIP2_HUMAN	Y	BCL2/adenovirus E1B 19 kDa protein-interacting protein 2	Cytoplasm	N	N
AVCDEPSEPEEEE<br>. ::: ..<br>EAYEMPSEEGYQD	194	1360	150	3	3	7	3	3	MSH6_HUMAN	N	DNA mismatch repair protein Msh6	Chromosome and Nucleus	Y	N
DLSDCPSEPLSDE<br>. . ::: .<br>EAYEMPSEEGYQD	211	1905	210	3	3	7	3	3	MTCL1_HUMAN	N	Microtubule cross-linking factor 1	Apical cell membrane, Cytoplasm, Lateral cell membrane and Midbody	N	Y #
RPKHRPSEADEEE<br>::: ..<br>EAYEMPSEEGYQD	652	1153	130	3	2	8	3	3	AP3D1_HUMAN	N	AP-3 complex subunit delta-1	Cytoplasm and Golgi apparatus membrane	Y	Y
NKFRTPSELHNDN<br>. ::: .<br>EAYEMPSEEGYQD	461	566	60	3	2	8	3	3	CSPG5_HUMAN	Y	Chondroitin sulfate proteoglycan 5	Cell junction, Cell membrane, Cell surface, Endoplasmic reticulum membrane and Golgi apparatus membrane	N	Y
GTKPPPSEGSDEE<br>::: ..<br>EAYEMPSEEGYQD	113	446	49	3	2	8	3	3	GRWD1_HUMAN	N	Glutamate-rich WD repeat-containing protein 1	Chromosome and Nucleus	N	N
RIFQVPSEMTEDI<br>.. :::<br>EAYEMPSEEGYQD	123	167	19	3	2	8	3	3	SPT19_HUMAN	Y	Spermatogenesis-associated protein 19, mitochondrial	Mitochondrion outer membrane	N	N

Supplementary Figure 14

c S87 human – part 1

Peptide alignment	Position	Length	MW list [kDa]	Exact	Similar	Mismatch	Max exact stretch	Max exact + similar stretch	ID	Also in mouse	Description	Subcellular location	Detected in plasma	Detected in CSF
TVEGAGSIAAATG<br>:::::::::::::<br>TVEGAGSIAAATG	81	140	14	13	0	0	13	13	SYUA_HUMAN	N	Alpha-synuclein	Cell junction. Cytoplasm. Membrane. Nucleus. Secreted	Y	Y
VDRGAGSIREAGG<br>::::: : :<br>TVEGAGSIAAATG	33	106	12	7	0	6	5	5	ATIF1_HUMAN	Y	ATPase inhibitor, mitochondrial	Mitochondrion	Y *	N
ADESTGSIAKRLQ<br>: ::::<br>TVEGAGSIAAATG	33	364	39	5	0	8	4	4	ALDOA_HUMAN	Y	Fructose-bisphosphate aldolase A	Cytoplasm	Y	Y
FDSLAGSIPATKV<br>:::: :<br>TVEGAGSIAAATG	13	593	66	5	0	8	4	4	CPNE5_HUMAN	Y	Copine-5	Cell projection. Perikaryon	N	N
ATVLSGSIAGLEG<br>:::: :<br>TVEGAGSIAAATG	981	2054	230	5	0	8	4	4	MY18A_HUMAN	Y	Unconventional myosin-XVIIIa	Cell surface. Cytoplasm. Endoplasmic reticulum-Golgi intermediate compartment. Golgi apparatus. Postsynaptic Golgi apparatus	Y	N
LLEEQGSIALRQE<br>: ::::<br>TVEGAGSIAAATG	1035	2472	280	5	0	8	4	4	SPTN1_HUMAN	Y	Spectrin alpha chain, non-erythrocytic 1	Cytoplasm	Y	Y
TEIRAGSISSEEV<br>: ::::<br>TVEGAGSIAAATG	1695	3051	330	5	0	8	4	4	BD1L1_HUMAN	N	Biorientation of chromosomes in cell division protein 1-like 1	Chromosome	N	N
AEDSDGSIASWEL<br>. ::::<br>TVEGAGSIAAATG	172	483	55	4	1	8	4	4	SMRD3_HUMAN	Y	SWI/SNF-related matrix-associated actin-dependent regulator of chromatin subfamily D member 3	Nucleus	N	N
ELSPAGSISKNSP<br>:::: .<br>TVEGAGSIAAATG	617	639	73	4	1	8	4	4	PARN_HUMAN	N	Poly(A)-specific ribonuclease PARN	Cytoplasm. Nucleus	N	N
HSPRAGSISPGSP<br>:::: .<br>TVEGAGSIAAATG	824	1409	150	4	1	8	4	4	TNS2_HUMAN	N	Tensin-2	Cell junction. Cell membrane	N	N
ALKEAGSIVRLYV<br>::::<br>TVEGAGSIAAATG	136	724	81	4	0	9	4	4	DLG4_HUMAN	Y	Disks large homolog 4	Cell junction. Cell membrane. Cell projection. Cytoplasm	N	Y
FSTFAGSITGPLY<br>::::<br>TVEGAGSIAAATG	412	916	100	4	0	9	4	4	NFM_HUMAN	N	Neurofilament medium polypeptide	Cell projection. Cytoplasm	Y	Y
IQARLGSIAEIDL<br>::::<br>TVEGAGSIAAATG	130	876	100	4	0	9	4	4	SRRT_HUMAN	Y	Serrate RNA effector molecule homolog	Cytoplasm. Nucleus	Y	Y #
QLNRAGSISTLDS<br>::::<br>TVEGAGSIAAATG	174	1216	130	4	0	9	4	4	RELCH_HUMAN	Y	RAB11-binding protein RELCH	Golgi apparatus. Recycling endosome	N	N
NLSEAGSIKKGER<br>::::<br>TVEGAGSIAAATG	198	1438	160	4	0	9	4	4	CLIP1_HUMAN	N	CAP-Gly domain-containing linker protein 1	Cell projection. Cytoplasm. Cytoplasmic vesicle membrane	Y	N
TVSCTGSIRYKTL<br>:: ::: :<br>TVEGAGSIAAATG	600	763	83	6	0	7	3	3	PLPR4_HUMAN	N	Phospholipid phosphatase-related protein type 4	Membrane	N	N
SASSEGSIHVAMG<br>. ::: : :<br>TVEGAGSIAAATG	164	1011	110	5	1	7	3	3	RASL3_HUMAN	N	RAS protein activator like-3	Cytoplasm	N	N
EVIRKGSITEYTA<br>: ::: :<br>TVEGAGSIAAATG	108	314	36	5	0	8	3	3	BNIP2_HUMAN	Y	BCL2/adenovirus E1B 19 kDa protein-interacting protein 2	Cytoplasm	N	N

Supplementary Figure 14

c **S87 human – part 2**

Peptide alignment	Position	Length	MW list [kDa]	Exact	Similar	Mismatch	Max exact stretch	Max exact + similar stretch	ID	Also in mouse	Description	Subcellular location	Detected in plasma	Detected in CSF
TALGGGSIRQSPL<br>: : :::<br>TVEGAGSIAAATG	228	503	51	5	0	8	3	3	WIPF1_HUMAN	N	WAS/WASL-interacting protein family member 1	Cell projection, Cytoplasm, Cytoplasmic vesicle	Y	N
AEEDNGSIGEETD<br>: ::: :<br>TVEGAGSIAAATG	654	685	77	5	0	8	3	3	STIM1_HUMAN	Y	Stromal interaction molecule 1	Cell membrane, Cytoplasm, Endoplasmic reticulum membrane, Sarcoplasmic reticulum	Y	Y
GNPGRGSIKTVAG<br>: ::: :<br>TVEGAGSIAAATG	164	979	110	5	0	8	3	3	UBP37_HUMAN	N	Ubiquitin carboxyl-terminal hydrolase 37	Unspecified	N	N
IIPHSGSIEKAEI<br>. ::: :<br>TVEGAGSIAAATG	219	372	42	4	1	8	3	3	ZN830_HUMAN	N	Zinc finger protein 830	Chromosome, Nucleus, Nucleus speckle	N	N
EEEEEGSIMNGST<br>: ::: .<br>TVEGAGSIAAATG	558	675	81	4	1	8	3	3	NEXN_HUMAN	Y	Nexilin	Cell junction and Cytoplasm	Y	N
ESQGKGSISEDEL<br>.: :::<br>TVEGAGSIAAATG	344	776	84	4	1	8	3	3	RTN1_HUMAN	N	Reticulon-1	Endoplasmic reticulum membrane, Golgi apparatus membrane	Y	Y
NKYGRGSISLNSS<br>: ::: .<br>TVEGAGSIAAATG	679	800	91	4	1	8	3	3	M3K20_HUMAN	N	Mitogen-activated protein kinase kinase kinase 20	Cytoplasm and Nucleus	N	N
SLSKEGSIGGSGG<br>. ::: :<br>TVEGAGSIAAATG	1108	1343	150	4	1	8	3	3	SYGP1_HUMAN	Y	Ras/Rap GTPase-activating protein SynGAP	Unspecified	N	N
SVATVGSICDLNL<br>.: :::<br>TVEGAGSIAAATG	2107	2602	280	4	1	8	3	3	FLNB_HUMAN	N	Filamin-B	Cytoplasm	Y	Y
MVLSRGSIFPMSV<br>: ::: .<br>TVEGAGSIAAATG	931	2701	310	4	1	8	3	3	ITPR2_HUMAN	Y	Inositol 1,4,5-trisphosphate receptor type 2	Endoplasmic reticulum membrane	N	N
GTDIGGSIRFPSS<br>. ::: .<br>TVEGAGSIAAATG	235	579	63	3	2	8	3	3	FAAH1_HUMAN	Y	Fatty-acid amide hydrolase 1	Cytoplasm and Endomembrane system	N	N
SGSHQGSIQELST<br>. ::: .<br>TVEGAGSIAAATG	546	630	71	3	2	8	3	3	KCND2_HUMAN	N	Potassium voltage-gated channel subfamily D member 2	Cell junction, Cell membrane, Cell projection, Perikaryon	N	N
ELQREGSIETLSN<br>. ::: .<br>TVEGAGSIAAATG	880	928	100	3	2	8	3	3	BCAS3_HUMAN	N	Breast carcinoma-amplified sequence 3	Cytoplasm and Nucleus	N	Y #
SRLSRGSISSTSE<br>. ::: .<br>TVEGAGSIAAATG	668	1821	210	3	2	8	3	3	PHIP_HUMAN	N	PH-interacting protein	Nucleus	N	N

Supplementary Figure 14

d Y39 human

Peptide alignment	Position	Length	MW list [kDa]	Exact	Similar	Mismatch	Max exact stretch	Max exact + similar stretch	ID	Also in mouse	Description	Subcellular location	Detected in plasma	Detected in CSF
EPDEILYVNMDEG<br>. .:::<br>TKEGVLYVGSKTK	815	894	98	3	2	8	3	4	UFO_HUMAN	Y	Tyrosine-protein kinase receptor UFO	Cell membrane	Y	Y
ASDGKLYVSSESR<br>.: ::: : ..<br>TKEGVLYVGSKTK	179	113<br>0	120	5	3	5	3	3	ABL1_HUMAN	Y	Tyrosine-protein kinase ABL1	Cytoplasm, Mitochondrion, Nucleus, Nuclear membrane	Y *	N
DHEEHLYVNTQGL<br>: ::: .<br>TKEGVLYVGSKTK	408	582	62	4	1	8	3	3	SHC2_HUMAN	Y	SHC-transforming protein 2	Unspecified	N	N
GGEDPLYVARRLV<br>: ::: .<br>TKEGVLYVGSKTK	528	665	72	4	1	8	3	3	WRIP1_HUMAN	Y	ATPase WRNIP1	Cytoplasm and Nucleus	N	N

e Y125 human

Peptide alignment	Position	Length	MW list [kDa]	Exact	Similar	Mismatch	Max exact stretch	Max exact + similar stretch	ID	Also in mouse	Description	Subcellular location	Detected in plasma	Detected in CSF
DPDNEAYEMPSEE<br>:::::::::::::<br>DPDNEAYEMPSEE	119	140	14	13	0	0	13	13	SYUA_HUMAN	Y	Alpha-synuclein	Cell junction, Cytoplasm, Membrane, Nucleus, Secreted	Y	Y
HYNGEAYEDDEHH<br>. ::::<br>DPDNEAYEMPSEE	375	397	45	4	1	8	4	4	DNJA1_HUMAN	Y	DnaJ homolog subfamily A member 1	Cytoplasm, Membrane, Microsome, Mitochondrion, Nucleus	Y	Y
YVDLQAYEDPAQG<br>: .::: : .<br>DPDNEAYEMPSEE	599	976	110	5	2	6	3	4	EPHA1_HUMAN	Y	Ephrin type-A receptor 1	Cell membrane	Y	N
DLEVPAYEDIFRD<br>: . ::: .<br>DPDNEAYEMPSEE	124	566	66	4	2	7	3	3	CDC45_HUMAN	N	Cell division control protein 45 homolog	Cytoplasm, Nucleus	N	N
FKIKNAYEESLDQ<br>::: ..<br>DPDNEAYEMPSEE	1488	1941	220	3	2	8	3	3	MYH2_HUMAN	N	Myosin-2	Cytoplasm	Y	Y #

f Y133 human

Peptide alignment	Position	Length	MW list [kDa]	Exact	Similar	Mismatch	Max exact stretch	Max exact + similar stretch	ID	Also in mouse	Description	Subcellular location	Detected in plasma	Detected in CSF
TPSAEGYQDVRDR<br>:: :::::<br>MPSEEGYQDYEPE	433	778	88	7	0	6	5	5	ABLM1_HUMAN	Y	Actin-binding LIM protein 1	Cytoplasm	Y	N
SAPTSGYQEFVHA<br>:::..<br>MPSEEGYQDYEPE	569	825	90	3	2	8	3	5	IL4RA_HUMAN	Y	Interleukin-4 receptor subunit alpha	Cell membrane, Secreted	Y	N
LLAKKGYQERDLE<br>. :::. . :<br>MPSEEGYQDYEPE	58	441	49	4	3	6	3	4	P2RX6_HUMAN	N	P2X purinoceptor 6	Membrane	N	N

Supplementary Figure 14

g Y136 human

Peptide alignment	Position	Length	MW list [kDa]	Exact	Similar	Mismatch	Max exact stretch	Max exact + similar stretch	ID	Also in mouse	Description	Subcellular location	Detected in plasma	Detected in CSF
GGYVQDYEDFM*<br>:::: :<br>EEGYQDYEPEA*	248	258	29	5	0	7	4	4	EIF3J_HUMAN	N	Eukaryotic translation initiation factor 3 subunit J	Cytoplasm	Y	Y
LEKIQDYEKMPE<br>: ::::<br>EEGYQDYEPEA*	490	569	65	5	0	7	4	4	IL1R1_HUMAN	Y	Interleukin-1 receptor type 1	Cell membrane, Membrane, Secreted	Y	Y
PVPNPDYEPIRK<br>::::<br>EEGYQDYEPEA*	182	207	23	4	0	8	4	4	CD3E_HUMAN	Y	T-cell surface glycoprotein CD3 epsilon chain	Cell membrane	N	N
MLRLQDYEEKTK<br>::::<br>EEGYQDYEPEA*	348	586	69	4	0	8	4	4	EZRI_HUMAN	Y	Ezrin	Apical cell membrane, Cell projection, Cytoplasm	Y	Y
EEDEEDYESSAK<br>:: .::: :<br>EEGYQDYEPEA*	64	1025	12<br>0	6	1	5	3	4	LCAP_HUMAN	Y	Leucyl-cystinyl aminopeptidase	Cell membrane and Secreted	Y	Y
VVGGEDYEEVDR<br>: .:::<br>EEGYQDYEPEA*	300	904	99	4	1	7	3	4	ZN598_HUMAN	N	E3 ubiquitin-protein ligase ZNF598	Unspecified	N	N
DDSDEDYEKVPL<br>.. .:::<br>EEGYQDYEPEA*	442	561	62	3	3	6	3	4	3BP2_HUMAN	Y	SH3 domain-binding protein 2	Unspecified	N	N
DLDDEDYEEDTP<br>. .::: .<br>EEGYQDYEPEA*	166	391	44	3	3	6	3	4	REQU_HUMAN	Y	Zinc finger protein ubi-d4	Cytoplasm and Nucleus	N	N
DDDEHDYEEILE<br>.. .:::<br>EEGYQDYEPEA*	626	643	72	3	3	6	3	4	THMS2_HUMAN	Y	Protein THEMIS2	Unspecified	N	N
PTDNEDYEHDDE<br>.::: .<br>EEGYQDYEPEA*	168	561	62	3	2	7	3	4	3BP2_HUMAN	Y	SH3 domain-binding protein 2	Unspecified	N	N
EEGAPDYENLQE<br>::: :::<br>EEGYQDYEPEA*	249	262	28	6	0	6	3	3	LAT_HUMAN	N	Linker for activation of T-cells family member 1	Cell membrane	Y	N
EELQDDYEDMME<br>:: :::<br>EEGYQDYEPEA*	2	911	10<br>0	5	0	7	3	3	B3AT_HUMAN	N	Band 3 anion transport protein	Basolateral cell membrane and Cell membrane	Y	Y
EEPEPDYEAIQT<br>:: :::<br>EEGYQDYEPEA*	381	432	47	5	0	7	3	3	PHAG1_HUMAN	Y	Phosphoprotein associated with glycosphingolipid-enriched microdomains 1	Cell membrane	Y*	Y
DEPEGDYEEVLE<br>.: :::<br>EEGYQDYEPEA*	391	486	54	4	1	7	3	3	HCLS1_HUMAN	Y	Hematopoietic lineage cell-specific protein	Cytoplasm, Membrane and Mitochondrion	Y	N
TSNFDDYEEEEI<br>. ::: :<br>EEGYQDYEPEA*	325	351	41	4	1	7	3	3	KAPCA_HUMAN	Y	cAMP-dependent protein kinase catalytic subunit alpha	Cell membrane, Cell projection, Cytoplasm, Cytoplasmic vesicle, Membrane, Mitochondrion and Nucleus	Y	N
TSNFDDYEEEDI<br>. ::: :<br>EEGYQDYEPEA*	325	351	41	4	1	7	3	3	KAPCB_HUMAN	N	cAMP-dependent protein kinase catalytic subunit beta	Cell membrane, Cytoplasm, Membrane and Nucleus	Y	N
SSKEDDYESDAA<br>::: .:<br>EEGYQDYEPEA*	364	5183	57<br>0	4	1	7	3	3	UBR4_HUMAN	Y	E3 ubiquitin-protein ligase UBR4	Cytoplasm, Membrane, Nucleus	Y	N
DPERSDYEEQQL<br>. ::: .<br>EEGYQDYEPEA*	65	394	44	3	2	7	3	3	ALKB5_HUMAN	N	RNA demethylase ALKBH5	Nucleus speckle	N	N
DDFDSDYENPDE<br>.. :::<br>EEGYQDYEPEA*	66	456	50	3	2	7	3	3	BLNK_HUMAN	Y	B-cell linker protein	Cell membrane and Cytoplasm	N	Y
DGPAVDYENQDV<br>. ::: .<br>EEGYQDYEPEA*	316	403	45	3	2	7	3	3	STAP2_HUMAN	N	Signal-transducing adaptor protein 2	Cytoplasm	N	N

Supplementary Figure 14. List of proteins that could cross-react with antibodies raised against human phosphorylated aSyn fragment (related to Figure 9)

a. Summary of the cross-reacting proteins that were previously detected in human plasma and Cerebrospinal fluid (CSF). **b-g**. For each fragment of 13 residues centred on a phosphorylated residue (**b:** S129, **c:** S87, **d:** Y39, **e:** Y125, **f:** Y133, **g:** Y136), the list of candidate hits is provided with local alignment against aSyn, starting position of hit alignment, length of the protein hit, its molecular weight, the number of exact matches, similar matches, and mismatches, the length of the largest continuous exact matches stretch and the length of the largest continuous exact or similar matches stretch. Uniprot ID, description and subcellular localization retrieved by parsing the "CC -!- SUBCELLULAR LOCATION:" fields of the corresponding Uniprot entries is also provided. Y- detected; N- not detected; Y*- detected by Proximity Extension Assay (PEA) technology; Y# - sequence coverage is reported as 0% in the CSF proteome resource database.

Supplementary Figure 15

a S129 mouse – part 1

Peptide alignment	Position	Length	MW list [kDa]	Exact	Similar	Mismatch	Max exact stretch	Max exact + similar stretch	ID	Also in human	Description	Subcellular location
EAYEMPSEEGYQD<br>:::::::::::::<br>EAYEMPSEEGYQD	123	140	14	13	0	0	13	13	SYUA_MOUSE	Y	Alpha-synuclein	Cell junction, Cytoplasm, Membrane, Nucleus and Secreted
KARENPSEEAQNL<br>: : ::::<br>EAYEMPSEEGYQD	115	501	59	6	0	7	4	4	SF3A3_MOUSE	Y	Splicing factor 3A subunit 3	Nucleus
LPEAPPSEEEEQE<br>:::: :.<br>EAYEMPSEEGYQD	810	1128	130	5	1	7	4	4	TEX2_MOUSE	Y	Testis-expressed protein 2	Endoplasmic reticulum membrane and Nucleus membrane
PPARRPSEEPEPD<br>:::: :<br>EAYEMPSEEGYQD	373	429	47	5	0	8	4	4	PHAG1_MOUSE	Y	Phosphoprotein associated with glycosphingolipid-enriched microdomains 1	Cell membrane
KIQRMPSESAAQS<br>:::: :<br>EAYEMPSEEGYQD	113	513	57	5	0	8	4	4	PLRG1_MOUSE	Y	Pleiotropic regulator 1	Nucleus and Nucleus speckle
IITEEPSEEEADM<br>: ::::<br>EAYEMPSEEGYQD	112	851	94	5	0	8	4	4	DDX21_MOUSE	Y	Nucleolar RNA helicase 2	Cytoplasm, Mitochondrion and Nucleus
SSPEQPSEEW***<br>: ::::<br>EAYEMPSEEGYQD	917	926	100	5	0	8	4	4	NIBA1_MOUSE	Y	Protein Niban 1	Cytoplasm and Membrane
PRESRPSEEREWD<br>:::: :<br>EAYEMPSEEGYQD	1153	1344	160	5	0	8	4	4	EIF3A_MOUSE	Y	Eukaryotic translation initiation factor 3 subunit A	Cytoplasm and Nucleus
ESGRSPSEEEEDA<br>: ::::<br>EAYEMPSEEGYQD	308	1693	190	5	0	8	4	4	ARG28_MOUSE	N	Rho guanine nucleotide exchange factor 28	Cell membrane and Cytoplasm
DIIRQPSEEEIIK<br>. ::::<br>EAYEMPSEEGYQD	110	130	15	4	1	8	4	4	PEA15_MOUSE	Y	Astrocytic phosphoprotein PEA-15	Cytoplasm
PDRTPPSEEDSAE<br>:::: .<br>EAYEMPSEEGYQD	79	315	34	4	1	8	4	4	SGTA_MOUSE	Y	Small glutamine-rich tetratricopeptide repeat-containing protein alpha	Cytoplasm and Nucleus
LVQHSPSEEKMSP<br>::::<br>EAYEMPSEEGYQD	140	262	29	4	0	9	4	4	EAF2_MOUSE	N	ELL-associated factor 2	Nucleus speckle
KELPPPSEEIKTG<br>::::<br>EAYEMPSEEGYQD	531	657	75	4	0	9	4	4	THOC1_MOUSE	Y	THO complex subunit 1	Cytoplasm, Nucleus, Nucleus matrix and Nucleus speckle
VTPGWPSEEEVGS<br>::::<br>EAYEMPSEEGYQD	447	733	79	4	0	9	4	4	CEP68_MOUSE	N	Centrosomal protein of 68 kDa	Cytoplasm
NGLSQPSEEEADI<br>::::<br>EAYEMPSEEGYQD	149	851	94	4	0	9	4	4	DDX21_MOUSE	Y	Nucleolar RNA helicase 2	Cytoplasm, Mitochondrion and Nucleus
NGLSQPSEEEVDI<br>::::<br>EAYEMPSEEGYQD	186	851	94	4	0	9	4	4	DDX21_MOUSE	Y	Nucleolar RNA helicase 2	Cytoplasm, Mitochondrion and Nucleus
AMLHLPSEQGTPE<br>.:::.: .<br>EAYEMPSEEGYQD	37	412	48	4	3	6	3	6	NEMO_MOUSE	Y	NF-kappa-B essential modulator	Cytoplasm and Nucleus
GTSELPSEDDGVE<br>:.:::. .<br>EAYEMPSEEGYQD	447	2323	260	4	3	6	3	6	C2CD3_MOUSE	N	C2 domain-containing protein 3	Cytoplasm

Supplementary Figure 15

a S129 mouse – part 2

Peptide alignment	Position	Length	MW list [kDa]	Exact	Similar	Mismatch	Max exact stretch	Max exact + similar stretch	ID	Also in human	Description	Subcellular location
EEAGNPSEDGMQS<br>: :::.: :<br>EAYEMPSEEGYQD	52	501	56	6	1	6	3	5	PWP1_MOUSE	N	Periodic tryptophan protein 1 homolog	Chromosome and Nucleus
ASRTLPSEDEEGE<br>.:::. .<br>EAYEMPSEEGYQD	8302	8799	1000	3	3	7	3	5	SYNE1_MOUSE	Y	Nesprin-1	Cytoplasm, Nucleus, Nucleus envelope, Nucleus outer membrane
DSSPLPSEKSDED<br>. .::: .:<br>EAYEMPSEEGYQD	1354	1711	200	4	3	6	3	4	CHD1_MOUSE	Y	Chromodomain-helicase-DNA-binding protein 1	Cytoplasm and Nucleus
LQPGGPSEDECSD<br>:::. :<br>EAYEMPSEEGYQD	263	332	37	4	1	8	3	4	PINX1_MOUSE	N	PIN2/TERF1-interacting telomerase inhibitor 1	Chromosome and Nucleus
ETQRKPSEDEKGF<br>: :::.<br>EAYEMPSEEGYQD	1055	1231	140	4	1	8	3	4	EHBP1_MOUSE	Y	EH domain-binding protein 1	Cytoplasm, Endosome and Membrane
YSVQEPSEDSEEE<br>. :::. ..<br>EAYEMPSEEGYQD	1169	1365	140	3	4	6	3	4	LMTK1_MOUSE	Y	Serine/threonine-protein kinase LMTK1	Cell projection, Cytoplasm and Membrane
QYNQVPSEDFERA<br>. . :::.<br>EAYEMPSEEGYQD	317	365	40	3	3	7	3	4	CXAR_MOUSE	N	Coxsackievirus and adenovirus receptor homolog	Basolateral cell membrane, Cell junction, Cell membrane and Secreted
DSSRLPSEGPQPA<br>. .:::<br>EAYEMPSEEGYQD	36	206	22	3	2	8	3	4	BLVRB_MOUSE	Y	Flavin reductase (NADPH)	Cytoplasm
RHQSDPSEDEDER<br>:::. .<br>EAYEMPSEEGYQD	123	496	57	3	2	8	3	4	SGK3_MOUSE	Y	Serine/threonine-protein kinase Sgk3	Cytoplasmic vesicle, Early endosome and Recycling endosome
DQQSLPSEPEEAL<br>. .:::<br>EAYEMPSEEGYQD	336	541	58	3	2	8	3	4	RETR2_MOUSE	N	Reticulophagy regulator 2	Membrane
KSVSTPSEAGSQD<br>::: : ::<br>EAYEMPSEEGYQD	85	612	68	6	0	7	3	3	DC1I2_MOUSE	N	Cytoplasmic dynein 1 intermediate chain 2	Cytoplasm
EEEEEPSEGVDEE<br>: : ::: ..<br>EAYEMPSEEGYQD	337	1044	120	5	2	6	3	3	GGYF1_MOUSE	Y	GRB10-interacting GYF protein 1	Unspecified
DLKESPSEGSLQP<br>. : ::: :<br>EAYEMPSEEGYQD	2	512	58	5	1	7	3	3	ASIC2_MOUSE	N	Acid-sensing ion channel 2	Cell membrane
PWSKRPSECGCEE<br>::: : ..<br>EAYEMPSEEGYQD	57	80	9	4	2	7	3	3	ADIG_MOUSE	N	Adipogenin	Membrane and Nucleus
KPGEEPSEYTDEE<br>: ::: ..<br>EAYEMPSEEGYQD	196	217	23	4	2	7	3	3	PGRC2_MOUSE	Y	Membrane-associated progesterone receptor component 2	Endoplasmic reticulum, Membrane and Nucleus envelope
EGLDTPSENSDEF<br>: . ::: .<br>EAYEMPSEEGYQD	83	326	38	4	2	7	3	3	BNIP2_MOUSE	Y	BCL2/adenovirus E1B 19 kDa protein-interacting protein 2	Cytoplasm
AKDEPPSEGEAEE<br>: ::: ..<br>EAYEMPSEEGYQD	467	543	62	4	2	7	3	3	NFL_MOUSE	Y	Neurofilament light polypeptide	Cell projection and Cytoplasm
PAERTPSEIQFHQ<br>: ::: ..<br>EAYEMPSEEGYQD	322	821	91	4	2	7	3	3	M4K2_MOUSE	Y	Mitogen-activated protein kinase kinase kinase kinase 2	Basolateral cell membrane, Cytoplasm and Golgi apparatus membrane
TVRYSPSEAGLHE<br>::: : ..<br>EAYEMPSEEGYQD	1829	2647	280	4	2	7	3	3	FLNA_MOUSE	Y	Filamin-A	Cell projection, Cytoplasm and Perikaryon

Supplementary Figure 15

a S129 mouse – part 3

Peptide alignment	Position	Length	MW list [kDa]	Exact	Similar	Mismatch	Max exact stretch	Max exact + similar stretch	ID	Also in human	Description	Subcellular location
EVRKKPSEGEEEA<br>: ::: .<br>EAYEMPSEEGYQD	162	192	21	4	1	8	3	3	CY24A_MOUSE	Y	Cytochrome b-245 light chain	Cell membrane
EDEGIPSENEEEK<br>: ::: .<br>EAYEMPSEEGYQD	2302	2776	300	4	1	8	3	3	AKP13_MOUSE	Y	A-kinase anchor protein 13	Cytoplasm, Membrane and Nucleus
AVCDEPSEPEEEE<br>. ::: ..<br>EAYEMPSEEGYQD	194	1358	150	3	3	7	3	3	MSH6_MOUSE	N	DNA mismatch repair protein Msh6	Chromosome and Nucleus
RIFQVPSEVMDDV<br>.. :::<br>EAYEMPSEEGYQD	110	154	18	3	2	8	3	3	SPT19_MOUSE	Y	Spermatogenesis-associated protein 19, mitochondrial	Mitochondrion outer membrane
FMKKAPSEIDPEE<br>::: ..<br>EAYEMPSEEGYQD	45	386	44	3	2	8	3	3	TTC4_MOUSE	N	Tetratricopeptide repeat protein 4	Cytoplasm and Nucleus
NKFRTPSELHNDN<br>. ::: .<br>EAYEMPSEEGYQD	461	566	60	3	2	8	3	3	CSPG5_MOUSE	Y	Chondroitin sulfate proteoglycan 5	Cell junction, Cell membrane, Cell surface, Endoplasmic reticulum membrane and Golgi apparatus membrane
LEDKAPSEVAIEE<br>::: ..<br>EAYEMPSEEGYQD	506	758	84	3	2	8	3	3	TDIF2_MOUSE	N	Deoxynucleotidyltransferase terminal-interacting protein 2	Nucleus
KRVRRPSESDKEE<br>::: ..<br>EAYEMPSEEGYQD	607	1684	180	3	2	8	3	3	AKA12_MOUSE	Y	A-kinase anchor protein 12	Cytoplasm. Membrane

Supplementary Figure 15

b S87 mouse– part 1

Peptide alignment	Position	Length	MW list [kDa]	Exact	Similar	Mismatch	Max exact stretch	Max exact + similar stretch	ID	Also in human	Description	Subcellular location
MDTGAGSIREAGG<br>::::: : :<br>TVEGAGSIAAATG	33	106	12	7	0	6	5	5	ATIF1_MOUSE	Y	ATPase inhibitor, mitochondrial	Mitochondrion
EPLGVGSIAAGGR<br>: :::::<br>TVEGAGSIAAATG	350	509	57	6	0	7	5	5	HARS1_MOUSE	N	Histidine--tRNA ligase, cytoplasmic	Cytoplasm
LLEEQGSIALRQG<br>: :::: :<br>TVEGAGSIAAATG	1035	2472	280	6	0	7	4	4	SPTN1_MOUSE	Y	Spectrin alpha chain, non-erythrocytic 1	Cytoplasm
ADESTGSIAKRLQ<br>: ::::<br>TVEGAGSIAAATG	33	364	39	5	0	8	4	4	ALDOA_MOUSE	Y	Fructose-bisphosphate aldolase A	Cytoplasm
FDSLAGSIPATKV<br>:::: :<br>TVEGAGSIAAATG	13	593	66	5	0	8	4	4	CPNE5_MOUSE	Y	Copine-5	Cell projection and Perikaryon
ATVLSGSIAGLEG<br>:::: :<br>TVEGAGSIAAATG	977	2050	230	5	0	8	4	4	MY18A_MOUSE	Y	Unconventional myosin-XVIIIa	Cytoplasm, Endoplasmic reticulum-Golgi intermediate compartment, Golgi apparatus and Postsynaptic Golgi apparatus
AEDSDGSIASWEL<br>. ::::<br>TVEGAGSIAAATG	172	483	55	4	1	8	4	4	SMRD3_MOUSE	Y	SWI/SNF-related matrix-associated actin-dependent regulator of chromatin subfamily D member 3	Nucleus
ALKEAGSIVRLYV<br>::::<br>TVEGAGSIAAATG	136	724	80	4	0	9	4	4	DLG4_MOUSE	Y	Disks large homolog 4	Cell junction, Cell membrane, Cell projection and Cytoplasm
QLNRAGSISTLDS<br>::::<br>TVEGAGSIAAATG	174	1216	130	4	0	9	4	4	RELCH_MOUSE	Y	RAB11-binding protein RELCH	Golgi apparatus and Recycling endosome
IQARLGSIAEIDL<br>::::<br>TVEGAGSIAAATG	130	875	100	4	0	9	4	4	SRRT_MOUSE	Y	Serrate RNA effector molecule homolog	Cytoplasm and Nucleus
RVERGGSISEASG<br>:: ::: :.:<br>TVEGAGSIAAATG	217	332	36	7	1	5	3	3	JSPR1_MOUSE	N	Junctional sarcoplasmic reticulum protein 1	Endoplasmic reticulum membrane and Sarcoplasmic reticulum membrane
TVTCTGSIRYKTL<br>:: ::: :<br>TVEGAGSIAAATG	602	766	83	6	0	7	3	3	PLPR4_MOUSE	N	Phospholipid phosphatase-related protein type 4	Membrane
EVIRKGSITEYTA<br>: ::: :<br>TVEGAGSIAAATG	108	326	38	5	0	8	3	3	BNIP2_MOUSE	Y	BCL2/adenovirus E1B 19 kDa protein-interacting protein 2	Cytoplasm
AEEDNGSIGEETD<br>: ::: :<br>TVEGAGSIAAATG	654	685	78	5	0	8	3	3	STIM1_MOUSE	Y	Stromal interaction molecule 1	Cell membrane, Cytoplasm, Endoplasmic reticulum membrane and Sarcoplasmic reticulum
LVPHSGSIEKAEI<br>: ::: :<br>TVEGAGSIAAATG	210	363	41	5	0	8	3	3	ZN830_MOUSE	N	Zinc finger protein 830	Chromosome, Nucleus and Nucleus speckle
SVSRRGSISSVSP<br>.: ::: .<br>TVEGAGSIAAATG	478	729	79	4	2	7	3	3	DTL_MOUSE	N	Denticleless protein homolog	Chromosome, Cytoplasm, Nucleus and Nucleus membrane
TPQRSGSISNYRS<br>: . :::<br>TVEGAGSIAAATG	490	559	64	4	1	8	3	3	AAPK1_MOUSE	N	5'-AMP-activated protein kinase catalytic subunit alpha-1	Cytoplasm and Nucleus
SVYFCGSIRGGRE<br>.: :::<br>TVEGAGSIAAATG	11	173	19	4	1	8	3	3	DNPH1_MOUSE	N	2'-deoxynucleoside 5'-phosphate N-hydrolase 1	Cytoplasm and Nucleus

Supplementary Figure 15

b S87 mouse – part 2

Peptide alignment	Position	Length	MW list [kDa]	Exact	Similar	Mismatch	Max exact stretch	Max exact + similar stretch	ID	Also in human	Description	Subcellular location
MVLSRGSIFPVSV<br>: ::: .<br>TVEGAGSIAAATG	931	2701	310	4	1	8	3	3	ITPR2_MOUSE	Y	Inositol 1,4,5-trisphosphate receptor type 2	Endoplasmic reticulum membrane
EEEEEGSIVNGST<br>: ::: .<br>TVEGAGSIAAATG	489	607	72	4	1	8	3	3	NEXN_MOUSE	Y	Nexilin	Cell junction and Cytoplasm
SLSKEGSIGGSGG<br>. ::: :<br>TVEGAGSIAAATG	1105	1340	150	4	1	8	3	3	SYGP1_MOUSE	Y	Ras/Rap GTPase-activating protein SynGAP	Cell junction and Membrane
EVDSLGSIHSIPS<br>:. :::<br>TVEGAGSIAAATG	340	898	110	4	1	8	3	3	TAOK3_MOUSE	N	Serine/threonine-protein kinase TAO3	Cytoplasm
ELQREGSIETLSN<br>. ::: .<br>TVEGAGSIAAATG	880	928	100	3	2	8	3	3	BCAS3_MOUSE	N	Breast carcinoma-amplified sequence 3 homolog	Cytoplasm and Nucleus
GTDIGGSIRFPSA<br>. ::: .<br>TVEGAGSIAAATG	235	579	63	3	2	8	3	3	FAAH1_MOUSE	Y	Fatty-acid amide hydrolase 1	Endoplasmic reticulum membrane and Golgi apparatus membrane

Supplementary Figure 15

c Y39 mouse

Peptide alignment	Position	Length	MW list [kDa]	Exact	Similar	Mismatch	Max exact stretch	Max exact + similar stretch	ID	Also in human	Description	Subcellular location
EPDEILYVNMDEG<br>. .:::<br>TKEGVLYVGSKTK	809	888	98	3	2	8	3	4	UFO_MOUSE	Y	Tyrosine-protein kinase receptor UFO	Cell membrane
ASDGKLYVSSESR<br>.: ::: : ..<br>TKEGVLYVGSKTK	179	1123	120	5	3	5	3	3	ABL1_MOUSE	Y	Tyrosine-protein kinase ABL1	Cytoplasm, Mitochondrion and Nucleus
KEVGGLYVASRAN<br>: ::: :.<br>TKEGVLYVGSKTK	302	1147	130	5	1	7	3	3	ASAP1_MOUSE	N	Arf-GAP with SH3 domain, ANK repeat and PH domain-containing protein 1	Cytoplasm and Membrane
TRPEPLYVNLALG<br>:. :::<br>TKEGVLYVGSKTK	1182	1305	140	4	1	8	3	3	RHG33_MOUSE	N	Rho GTPase-activating protein 33	Cell membrane
DYEEHLYVNTQGL<br>: ::: .<br>TKEGVLYVGSKTK	389	573	62	4	1	8	3	3	SHC2_MOUSE	Y	SHC-transforming protein 2	Unspecified
GGEDPLYVARRLV<br>: ::: .<br>TKEGVLYVGSKTK	523	660	72	4	1	8	3	3	WRIP1_MOUSE	Y	ATPase WRNIP1	Cytoplasm and Nucleus

d Y125 mouse

Peptide alignment	Position	Length	MW list [kDa]	Exact	Similar	Mismatch	Max exact stretch	Max exact + similar stretch	ID	Also in human	Description	Subcellular location
DPGSEAYEMPSEE<br>:: :::::::::<br>DPDNEAYEMPSEE	119	140	14	11	0	2	9	9	SYUA_MOUSE	Y	Alpha-synuclein	Cell junction, Cytoplasm, Membrane, Nucleus and Secreted
EEEGEAYEEPDSE<br>. . :::: : :<br>DPDNEAYEMPSEE	396	547	60	6	2	5	4	4	CD19_MOUSE	N	B-lymphocyte antigen CD19	Cell membrane and Membrane raft
HYNGEAYEDDEHH<br>. ::::<br>DPDNEAYEMPSEE	375	397	45	4	1	8	4	4	DNJA1_MOUSE	Y	DnaJ homolog subfamily A member 1	Cytoplasm, Membrane, Microsome, Mitochondrion and Nucleus
YVDLQAYEDPAQG<br>: .::: : .<br>DPDNEAYEMPSEE	600	977	110	5	2	6	3	4	EPHA1_MOUSE	Y	Ephrin type-A receptor 1	Cell membrane
SSGPDAYEEVTDG<br>.::: ..<br>DPDNEAYEMPSEE	413	453	50	3	3	7	3	4	PTN18_MOUSE	N	Tyrosine-protein phosphatase non-receptor type 18	Cytoplasm and Nucleus
FKVKNAYEESLDQ<br>::: ..<br>DPDNEAYEMPSEE	1486	1939	220	3	2	8	3	3	MYH4_MOUSE	N	Myosin-4	Cytoplasm

Supplementary Figure 15

e Y133 mouse

Peptide alignment	Position	Length	MW list [kDa]	Exact	Similar	Mismatch	Max exact stretch	Max exact + similar stretch	ID	Also in human	Description	Subcellular location
TPSAEGYQDVRDR<br>:: :::::<br>MPSEEGYQDYEPE	477	861	97	7	0	6	5	5	ABLM1_MOUSE	Y	Actin-binding LIM protein 1	Cytoplasm
PAPAGGYQEFVQA<br>:::..<br>MPSEEGYQDYEPE	569	810	88	3	2	8	3	5	IL4RA_MOUSE	Y	Interleukin-4 receptor subunit alpha	Cell membrane and Secreted
MVTFQGYQKQTRW<br>: . .:::<br>MPSEEGYQDYEPE	437	575	64	4	2	7	3	4	I10R1_MOUSE	N	Interleukin-10 receptor subunit alpha	Cell membrane and Cytoplasm

f Y136 mouse

Peptide alignment	Position	Length	MW list [kDa]	Exact	Similar	Mismatch	Max exact stretch	Max exact + similar stretch	ID	Also in human	Description	Subcellular location
GGYVQDYEDFM*<br>:::: :<br>EEGYQDYEPEA*	253	263	29	5	0	7	4	4	EI3JB_MOUSE	N	Eukaryotic translation initiation factor 3 subunit J-B	Cytoplasm
LEKIQDYEKMPD<br>: ::::<br>EEGYQDYEPEA*	493	576	67	5	0	7	4	4	IL1R1_MOUSE	Y	Interleukin-1 receptor type 1	Cell membrane, Membrane and Secreted
PVPNPDYEPIRK<br>::::<br>EEGYQDYEPEA*	164	189	21	4	0	8	4	4	CD3E_MOUSE	Y	T-cell surface glycoprotein CD3 epsilon chain	Cell membrane
MLRLQDYEQKTK<br>::::<br>EEGYQDYEPEA*	348	586	69	4	0	8	4	4	EZRI_MOUSE	Y	Ezrin	Apical cell membrane, Cell projection and Cytoplasm
DEDEEDYESSAK<br>.: .::: :<br>EEGYQDYEPEA*	64	1025	120	5	2	5	3	4	LCAP_MOUSE	Y	Leucyl-cystinyl aminopeptidase	Cell membrane and Endomembrane system
EDSDEDYEKVPL<br>:. .:::<br>EEGYQDYEPEA*	440	559	62	4	2	6	3	4	3BP2_MOUSE	Y	SH3 domain-binding protein 2	Unspecified
DMDDHDYEEI**<br>. .::: :<br>EEGYQDYEPEA*	654	663	74	4	2	6	3	4	THMS2_MOUSE	Y	Protein THEMIS2	Unspecified
PMDNEDYEHEDE<br>.::: :<br>EEGYQDYEPEA*	168	559	62	4	1	7	3	4	3BP2_MOUSE	Y	SH3 domain-binding protein 2	Unspecified
DLDDEDYEEDTP<br>. .::: .<br>EEGYQDYEPEA*	166	391	44	3	3	6	3	4	REQU_MOUSE	Y	Zinc finger protein ubi-d4	Cytoplasm and Nucleus
ERGRSDYESVGS<br>: : :::<br>EEGYQDYEPEA*	97	660	73	5	0	7	3	3	DDX3L_MOUSE	N	Putative ATP-dependent RNA helicase PI10	Unspecified
EEPEPDYEAIQT<br>:: :::<br>EEGYQDYEPEA*	380	429	47	5	0	7	3	3	PHAG1_MOUSE	Y	Phosphoprotein associated with glycosphingolipid-enriched microdomains 1	Cell membrane
EDAEGDYEDVLE<br>:. :::<br>EEGYQDYEPEA*	399	486	54	4	1	7	3	3	HCLS1_MOUSE	Y	Hematopoietic lineage cell-specific protein	Mitochondrion
TSNFDDYEEEEI<br>. ::: :<br>EEGYQDYEPEA*	325	351	41	4	1	7	3	3	KAPCA_MOUSE	Y	cAMP-dependent protein kinase catalytic subunit alpha	Cell membrane, Cell projection, Cytoplasm, Cytoplasmic vesicle, Membrane, Mitochondrion and Nucleus
ESWRNDYEEDDL<br>: ::: .<br>EEGYQDYEPEA*	138	853	97	4	1	7	3	3	TGS1_MOUSE	N	Trimethylguanosine synthase	Cytoplasm and Nucleus
SSKEDDYESDAA<br>::: .:<br>EEGYQDYEPEA*	364	5180	570	4	1	7	3	3	UBR4_MOUSE	Y	E3 ubiquitin-protein ligase UBR4	Cytoplasm, Membrane, Nucleus
DDFDSDYENPDE<br>.. :::<br>EEGYQDYEPEA*	66	457	51	3	2	7	3	3	BLNK_MOUSE	Y	B-cell linker protein	Cell membrane and Cytoplasm

Supplementary Figure 15. List of proteins that could cross-react with antibodies raised against mouse phosphorylated aSyn fragment (related to Figure 9)

a-f. For each fragment of 13 residues centered on a phosphorylated residue (**a:** S129, **b:** S87, **c:** Y39, **d:** Y125, **e:** Y133, **f:** Y136), the list of candidate hits is provided with local alignment against aSyn, starting position of hit alignment, length of the protein hit, its molecular weight, the number of exact matches, similar matches, and mismatches, the length of the largest continuous exact matches stretch and the length of the largest continuous exact or similar matches stretch. Uniprot ID, description and subcellular localization retrieved by parsing the "CC -!- SUBCELLULAR LOCATION:" fields of the corresponding Uniprot entries is also provided.

Supplementary Figure 16

a

				pSyn #64	EP 1536Y	MJF-R13	81A	GTX	pS129-LASH-EGT	Figures
KO mice	Primary neurons	ICC	Neurites	+	+/-	+	+	+/-	+	Figure S8
			Cell bodies	+	+/-	+	+	+/-	+	Figure S8
			Nucleus	+	-	+	-	-	+	Figure S8
		WB	HMWs > 25 kDa	+	+	+	+	-	-	Figure 5
			17 kDa	+	+	+	+	+	-	Figure 5
			15 kDa	-	-	-	-	-	-	Figure 5
			< 15 kDa	-	-	-	-	-	-	Figure 5
	Mouse tissues	IHC	Neurites	-	-	-	+	+/-	-	Figure 7A
			Cell bodies	+/-	+/-	+	+	-	-	Figure 7A
			Nucleus	+	-	+	-	-	+/-	Figure 7A
		WB	HMWs > 25 kDa	+	-	++	-	-	+/-	Figure 7B and S12
			17 kDa	+	+/-	+	-	-	-	Figure 7B and S12
			15 kDa	-	-	-	-	-	-	Figure 7B and S12
			< 15 kDa	-	-	-	-	-	-	Figure 7B and S12

Supplementary Figure 16

b

				pSyn #64	EP 1536Y	MJF-R13	81A	GTX	pS129-LASH-EGT	Figures
WT mice	PFFs-treated Primary neurons	ICC	Neurites	+	+	+	+	+	+	Figures 4A, 6 and S6-S7
			Cell bodies	+	+	+	+	+	+	Figures 4A, 6 and S6-S7
			Nucleus	+	-	+	-	-	-	Figure 6
			Morphological diversity	-	Not tested	+	-	+	Not tested	Figures 6 and S7
		HTS	Background	++	+/-	++	+/-	+/-	+/-	Figures 4B-C and S6A
		WB	HMWs > 25 kDa	+	-	++	+	-	-	Figures 4D and S6B
			17 kDa (unspecific)*	+	+	+	+	+	+	Figures 4D and S6B
			15 kDa	+	+/-	++	+	-	-	Figures 4A, 6 and S6-S7
			< 15 kDa (unspecific)*	+	+	+	+	+	+	Figures 4A, 6 and S6-S7
	PFFs-injected Mouse tissues	IHC	Neurites	+	++	+	++	+/-	+	Figure 8A and B
			Cell bodies	++	++	+	+/-	+	++	Figure 8A and B
			Nucleus	+	++	-	+/-	+/-	+	Figure 8A and B
		WB	HMWs > 25 kDa	N.D	N.D	N.D	N.D	N.D	N.D	
			17 kDa	N.D	N.D	N.D	N.D	N.D	N.D	
			15 kDa	N.D	N.D	N.D	N.D	N.D	N.D	
			< 15 kDa	N.D	N.D	N.D	N.D	N.D	N.D	

Supplementary Figure 16. pS219 background and specificity in the neuronal and animal seeding models (related to Figures 2-8)

a-b. Tables recapitulating our main observations regarding the background and specificity of the six pS129 antibodies (pSyn#64, MJF-R13, 81A, EP1536Y, GTX and pS129-LASH-EGT) for ICC, HTS, IHC and WB applications in aSyn KO or PFFs-treated WT primary neurons or mice. * Detected both in the PBS- and the PFF-treated neurons. N.D for non-determined.

Videos 1 and 2: 3D animations of the “nuclear-like” pS129-aSyn aggregates inside the nucleus.

The “nuclear-like” confocal images depicted in Figure 5 were processed using Imaris software. The resulting 3D animations showing the nuclear surface's interior clearly demonstrate that the “nuclear-like” pS129-aSyn aggregates are found within the nuclear volume.

Videos 1 and 2 correspond to the “nuclear-like” pS129-aSyn aggregates stained with the MJF-R-13 or the GTX antibodies, respectively.
